# Environmentally driven immune imprinting protects against allergy

**DOI:** 10.1038/s41586-025-10001-5

**Published:** 2026-01-28

**Authors:** S. Erickson, B. Lauring, J. Cullen, R. Medzhitov

**Affiliations:** 1 Department of Immunobiology, Yale University School of Medicine, New Haven, CT USA; 2 Tananbaum Center for Theoretical and Analytical Human Biology, Yale University School of Medicine, New Haven, CT USA; 3 Howard Hughes Medical Institute, Chevy Chase, MD USA

**Keywords:** Immunology, Adaptive immunity

## Abstract

Allergic diseases are caused by overexuberant type II immune responses mounted against environmental antigens^1^. The allergic state is typified by the presence of allergen-reactive immunoglobulin E (IgE), which triggers mast cell degranulation upon allergen encounter, manifesting in pruritis, oedema and, in severe cases, anaphylaxis. Over the past century, the prevalence of allergic diseases has increased markedly, suggesting that environmental rather than genetic factors are mediating this change^2^. Although many hypotheses connecting environment to allergy exist^3–6^, the biological mechanisms that underpin environmentally mediated protection from allergy are unknown. Here we show, using a mouse model of allergic disease, that exposure to immunostimulatory environments generated cross-reactive adaptive immune memory, which tracked with obstructed type II immune responses upon allergen exposure. We found that engagement of cross-reactive adaptive immunity protected against future allergic sensitization and suppressed established allergic responses. Cross-reactivity in a tolerogenic context also prevented allergy, with the effect extending across antigenically complex exposures even at low protein sequence similarity. Our findings demonstrate a mechanistic relationship between environment and allergy, with general implications for adaptive immune function in natural settings.

## Abstract (web summary)

In a mouse model, environmental immunostimulation in early life led to cross-reactive adaptive immune memory and reduced type II immune responses to allergens, indicating a mechanistic relationship between environmental antigen exposure and subsequent allergy.

## Main

The adaptive immune system couples antigen recognition with distinct effector functions for efficient control of diverse foreign agents. Viral and bacterial infections induce effector functions that are collectively referred to as type I immune responses, whereas type II immune responses are typically elicited through exposure to parasites, irritants and toxins^7^. Allergic diseases, including asthma, atopic dermatitis and food allergy, are considered to be exaggerated type II immune responses mounted against otherwise innocuous or mild insults. Although underlying genetic factors determine individual predisposition towards allergy development, epidemiological evidence suggests that environmental changes are driving the recent rise in allergic diseases^3,8,9^. The evidence for environmental influence on allergy in humans may be summarized in three observations: (1) that the prevalence of allergic disease has a nonuniform distribution across the world; (2) that individuals from similar genetic backgrounds have different rates of allergy regionally; and (3) that the incidence of allergies has increased rapidly over the past century, at a pace that cannot be explained by genetics alone^2,10–14^.

Most, but not all, cases of allergic disease initiate during infancy or childhood, and the allergic state persists for an extended period, often the entire lifespan of the individual^15^. Therapeutic interventions may provide desensitization towards specific allergens, but do not durably alter the underlying allergic immune setpoint, suggesting that allergic or non-allergic immune states are established upon initial allergen encounters and are maintained thereafter^16,17^. Thus, environmental effects that influence polarization towards either opposing allergic or non-allergic states are likely to occur before or during primary exposure to a novel allergen. The present study was undertaken to identify, in a mouse model of allergic sensitization, environmental factors relevant to protection from allergic disease.

## Pet shop mice are protected from allergy

**Extended Data Fig. 1:**
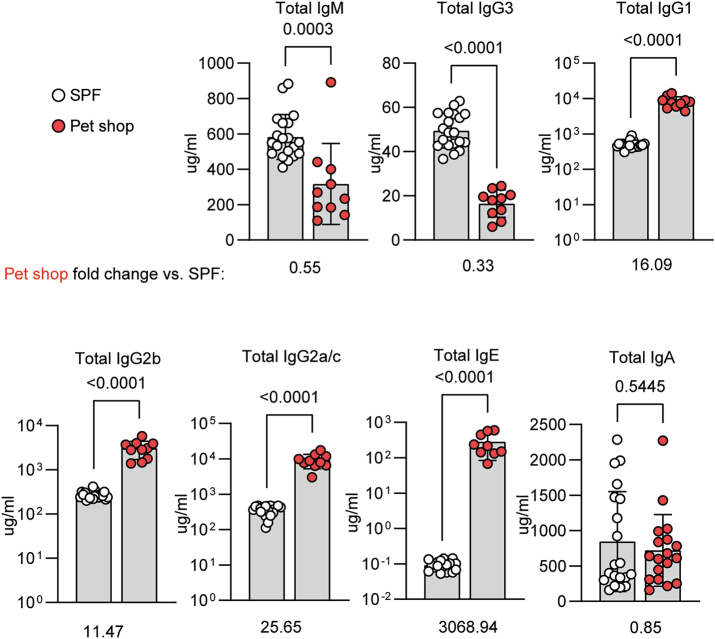
Baseline serum antibodies in SPF and pet shop mice. Baseline serum immunoglobulin levels in adult SPF (BALB/cJ) and pet shop mice (n = 20 SPF, 10 pet shop). Isotypes ordered by locus position. Statistical significance was determined using two-tailed unpaired Student’s t-test. Error bars represent SD. Data are pooled from three separate cohorts of mice obtained over the span of six months. Source data

Previous studies have shown that the immune systems of mice raised in specific pathogen-free (SPF) conditions are similar to those of neonatal humans, whereas the immunological profiles of mice raised outside laboratory settings or collected from the wild resemble those of healthy adult humans^18,19^. Because adult humans, unlike infants or SPF mice, are generally protected from allergic sensitization, we explored whether non-SPF mice would also be protected from developing allergies. Outbred non-SPF mice were procured from a local breeder (hereafter referred to as pet shop mice). Pet shop mice were subjected to pathogen testing and faecal microbiota profiling upon their arrival in laboratory facilities and were found to harbour a variety of pathogens and commensal bacteria (Supplementary Table 1 and Supplementary Fig. 2). Accordingly, pet shop mice had serological profiles that were consistent with the diverse pathogen and microbial exposure typically seen in non-SPF mice^18,20,21^ (Extended Data Fig. 1).

**Fig. 1:**
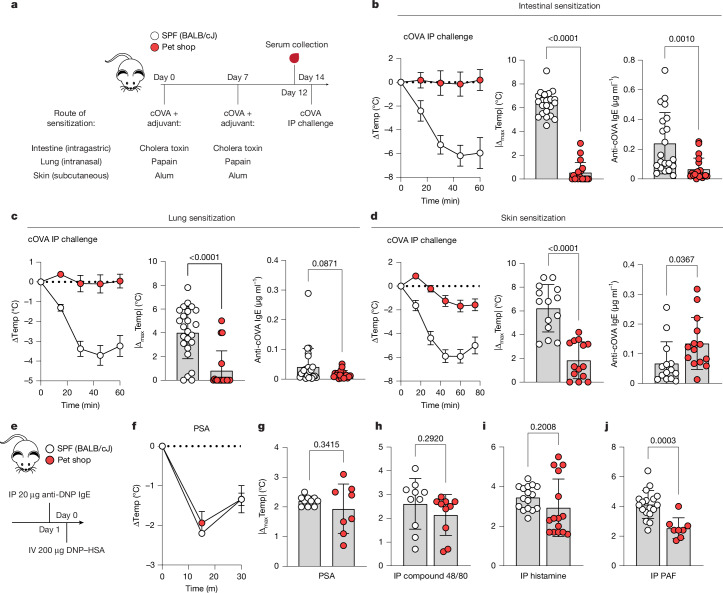
Pet shop mice are protected from allergen-induced anaphylaxis. **a**, Experimental scheme for induction of allergic sensitization. SPF (BALB/cJ) and pet shop mice were administered cOVA by the indicated route with adjuvant on day 0 and day 7, serum was collected on day 12, and mice were challenged intraperitoneally (IP) with cOVA on day 14. **b**–**d**, Core temperature (Temp) following systemic cOVA challenge (left), maximal temperature decrease (middle) and cOVA-reactive IgE levels (right) following intestinal sensitization (**b**; SPF: *n* = 22; pet shop: *n* = 19), lung sensitization (**c**; SPF: *n* = 24; pet shop: *n* = 18) or skin sensitization (**d**; *n* = 14). **e**, Experimental scheme for IgE-mediated passive systemic anaphylaxis (PSA). IV, intravenous. **f**,**g**, Kinetics of temperature decrease (**f**) and maximal temperature decrease (**g**) following challenge with dinitrophenyl–human serum albumin (DNP–HSA) (SPF: *n* = 10; pet shop: *n* = 8). **h**–**j**, Maximal temperature decrease following intraperitoneal injection of mast cell secretagogue compound 48/80 (**h**) (*n* = 10), histamine dihydrochloride (**i**) (SPF: *n* = 16; pet shop: *n* = 15) or PAF-16 (**j**) (SPF: *n* = 18; pet shop: *n* = 8). Data are pooled from two or more repeats of each experiment. Source data

To test allergic sensitization potential, SPF and pet shop mice were exposed to a model allergen—chicken ovalbumin (cOVA)—at three barrier tissue sites with type II-driving adjuvants, and then assayed for anaphylactic responses to systemic cOVA exposure^22^ (Fig. 1a). Notably, and regardless of sensitization route, pet shop mice were only mildly responsive to allergen challenge, whereas SPF mice underwent severe anaphylactic shock and hypothermia (Fig. 1b–d, left). As a measure of allergic sensitivity, the absolute maximum amount of body temperature lost was calculated for each mouse following systemic challenge (Fig. 1b–d, middle). Pet shop mice produced less antigen-reactive IgE than SPF mice after intestinal or lung exposure, but produced equal or greater amounts of anti-cOVA IgE following subcutaneous exposure (Fig. 1b–d, right). Whereas the protection from anaphylaxis following intestinal or lung exposure may be simply explained by reduced production of IgE, the skin sensitization data suggested that some obstruction of IgE-mediated mast cell activation or an impaired response to mast cell-derived anaphylactogens might be present in the system. To test protection from anaphylaxis at the effector level, we subjected SPF and pet shop mice to passive IgE-dependent or IgE-independent mast cell activation, observing no difference between groups (Fig. 1e–h). Direct provision of the anaphylactogens histamine and platelet activating factor (PAF) showed that pet shop mice had similar, although generally slightly reduced, responses to signals downstream of effector cells (Fig. 1i, j). Therefore, the subdued allergic response seen in pet shop mice after sensitization was not fully accounted for by global defects in anaphylactic potential.

**Extended Data Fig. 2:**
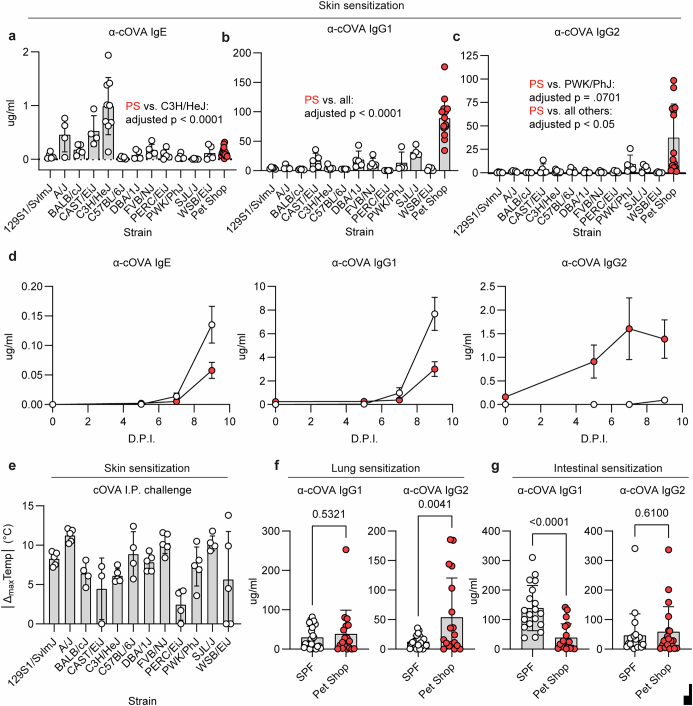
Pet shop mice mount unusual antibody responses after sensitization. (**a-c**) Comparison of antigen-reactive IgE (**a**), IgG1 (**b**) and IgG2 (**c**) following skin sensitization in the indicated inbred SPF strains and pet shop mice (n = 14 pet shop, 10 C3H/HeJ, 5 others). (**d**) Kinetics of antibody production by isotype following primary sensitization in SPF (BALB/cJ) and pet shop mice (n = 30 SPF, 31 pet shop). (**e**) Maximal temperature loss following systemic cOVA challenge in the indicated strains (n = 3 CAST/EiJ, 4 (BALB/cJ, C57BL/6 J, PERC/EiJ), 5 others). (**f-g**) Antigen-reactive antibody levels of the indicated isotype following lung (n = 24 SPF, 18 pet shop) (**f**) or intestinal (n = 21 SPF, 20 pet shop) (**g**) sensitization. Statistical significance was determined using one-way ANOVA with Tukey correction for multiple comparisons (**a-c**) or two-tailed unpaired Student’s t-test (**e,f**). Error bars represent SD. Data are pooled from two or more repeats of each experiment. Source data

**Extended Data Fig. 3:**
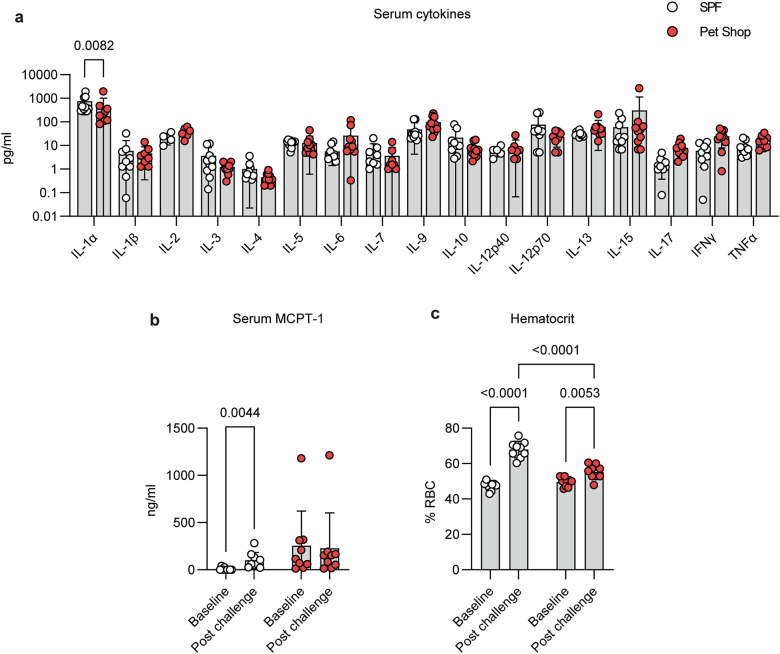
Baseline serum cytokines and responses to allergen challenge. (**a**) Quantification of cytokines in serum taken at baseline from adult SPF (BALB/cJ) or pet shop mice (n = 10). (**b**) serum MCPT-1 levels in SPF and pet shop mice before and 60 min after allergen challenge (n = 9). (**c**) Hematocrit of SPF and pet shop mice before and 60 min after allergen challenge (n = 9). Statistical significance was determined using two-way ANOVA with Šidák correction for multiple comparisons (**a,c**) or one-way ANOVA with Tukey correction for multiple comparisons (**b**). Error bars represent SD. Data are pooled from two or more repeats of each experiment except (**a**), which was performed once. Source data

On the basis of these results, we reasoned that protection from active anaphylactic responses may occur upstream of mast cell activation in pet shop mice. Whereas pet shop mice elaborated allergen-reactive IgE levels in line with several inbred SPF strains following skin sensitization, the levels of allergen-reactive IgG1 and IgG2 in pet shop mice were highly elevated, with IgG2 displaying altered kinetics (Extended Data Fig. 2a–d). Although not all inbred SPF strains were equally susceptible to anaphylaxis (Extended Data Fig. 2e), none resembled pet shop mice serologically, highlighting that background genetics alone were unlikely to explain the humoral response in pet shop mice. Across other routes of sensitization, pet shop mice also produced a higher allergen-reactive IgG:IgE ratio than inbred SPF mice (Extended Data Fig. 2f,g). In line with a previous report^23^, we did not detect elevated serum cytokines at baseline that would influence production of IgG versus IgE, although this might not account for cytokines in the lymph node microenvironment (Extended Data Fig. 3a). We did notice that, at baseline, pet shop mice have appreciable serum mast cell protease 1 (MCPT-1) indicative of ongoing mast cell activation, but MCPT-1 was not increased following anaphylactic challenge (Extended Data Fig. 3b). As further confirmation of protection from anaphylaxis, pet shop mice had a marginal increase in haematocrit following challenge, suggesting that we were not missing anaphylactic events by temperature readouts (Extended Data Fig. 3c).

**Extended Data Fig. 4:**
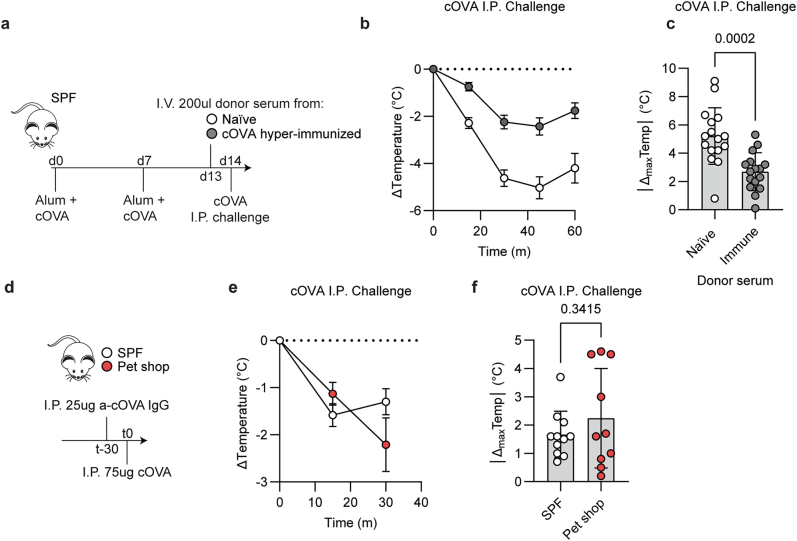
Passive transfer of antigen-reactive IgG in protecting from or driving anaphylaxis. (**a**) Experimental scheme to measure IgG-mediated protection from anaphylaxis. Heat-treated sera from naïve or (CFA-adjuvanted) cOVA-immunized SPF (BALB/cJ, approximately 800ug/ml cOVA-reactive IgG) mice were transferred to cOVA-sensitized animals one day prior to systemic cOVA challenge. (**b-c**) Core body temperature measurements (**b**) and maximal temperature loss (**c**) following systemic cOVA challenge (n = 16). (**d**) experimental scheme to test IgG-mediated passive anaphylaxis in SPF and pet shop mice. Animals received 25ug polyclonal cOVA-reactive IgG thirty minutes prior to systemic challenge with cOVA. (**e-f**) Core body temperature measurements (**e**) and maximal temperature loss (**f**) following challenge (n = 11 SPF, 10 pet shop). Statistical significance was determined with two-tailed unpaired Student’s t-test (**c**). Error bars represent SEM (**b**) or SD (**c**). Data are pooled from two or more repeats of each experiment. Source data

High levels of allergen-reactive IgG are thought to be the protective mechanism of allergen immunotherapy by binding allergen and preventing engagement with IgE-coated mast cells or by inhibiting mast cell activation via the inhibitory Fc receptor FcRγIIb^24–26^. Transfer of allergen-reactive IgG-rich serum to allergic SPF mice was sufficient to reduce subsequent anaphylaxis, consistent with the notion that the high level of allergen-reactive IgG seen in pet shop mice can be protective (Extended Data Fig. 4a–c). IgG-mediated anaphylaxis can also occur, typically at lower concentrations of antibody than we used in testing passive protection. Administering a smaller dose of antigen-reactive IgG immediately before systemic challenge showed that IgG-mediated anaphylaxis in SPF and pet shop mice was equivalent (Extended Data Fig. 4d–f). Together, these data suggested that pet shop mice, unlike SPF mice, are protected from allergic sensitization by mounting IgG-dominant humoral responses to allergen exposure.

## Pet shop mice have prior immune memory

**Fig. 2:**
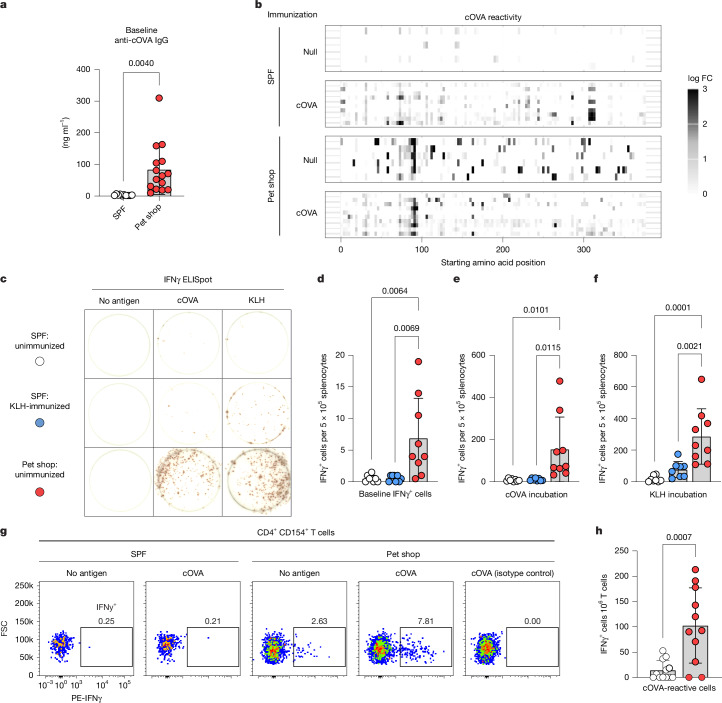
Pet shop mice have pre-existing immune memory of model antigens. **a**, cOVA-reactive IgG from sera of unimmunized SPF (C57BL/6J) or pet shop mice (SPF: *n* = 10; pet shop: *n* = 15) at 1:100 dilution. **b**, Epitope profiling of cOVA-reactive IgG in SPF (C57BL/6J) and pet shop mice with or without cOVA exposure (*n* = 6 baseline; 10 immunized). Each row represents one sample. FC, fold change. **c**, Representative ELISpot images of IFNγ^+^ splenocytes from the indicated sources incubated without antigen or with cOVA or KLH. **d**–**f**, Quantification of IFNγ^+^ spots at baseline (**d**) and after incubation with cOVA (**e**) or KLH (**f**) (SPF unimmunized: *n* = 8; SPF KLH: *n* = 8; pet shop unimmunized: *n* = 9). Colours as indicated in **c**. **g**, Representative flow cytometry plots of CD4^+^CD154^+^ T cells from the indicated sources following incubation with or without cOVA. **h**, Background-corrected quantification of IFNγ^+^CD4^+^ T cells after incubation with cOVA (SPF: *n* = 11; pet shop: *n* = 12). Two-tailed unpaired Student’s *t*-test (**a**,**h**) or one-way ANOVA with Tukey correction for multiple comparisons (**d**–**f**). Error bars represent s.d. Data are pooled from two or more repeats of each experiment or representative of two experiments (**b**). Source data

**Extended Data Fig. 5:**
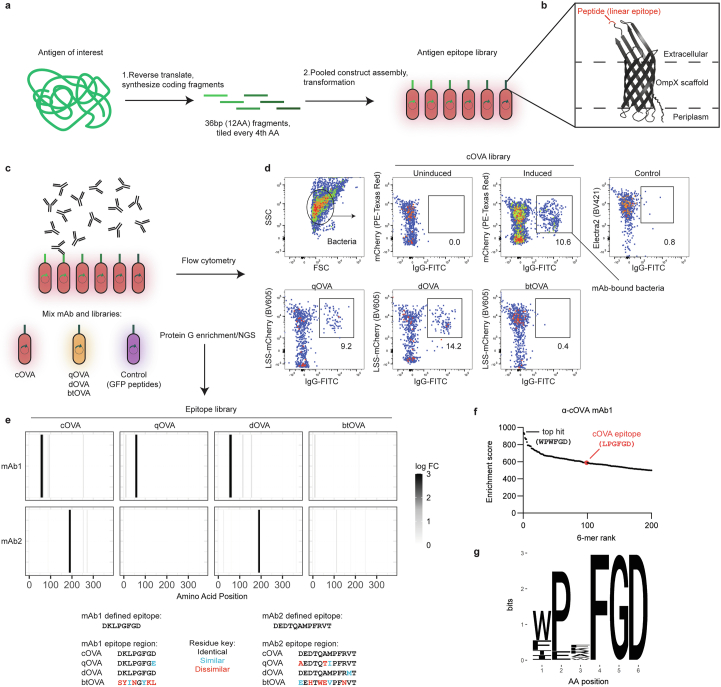
A bacterial epitope display system for OVA antibody reactivity profiling. (**a**) Creation of peptide epitope libraries for serological testing. Amino acid sequences of antigens were reverse translated, segmented into overlapping 36 bp tiles and cloned en masse into a display scaffold plasmid also coding for one of three fluorescent proteins. (**b**) Cartoon depiction of the eCPX scaffold on the bacterial surface, with displayed peptide in red. (**c-e**) The libraries were mixed with mAbs of known reactivities and screened by flow cytometry for binding (**d**) or protein G-enriched for sequencing (**e**). (**f**) Top 200 6mer epitopes enriched by OVA mAb1 from a universal epitope library. (**g**) Sequence logo of the top ten 6mer epitopes enriched by mAb1. Data are representative of two or more separate experiments. Source data

Protection from the allergic state in pet shop mice correlated with alterations in the adaptive immune response towards allergens. Surprisingly, pet shop mice had detectable cOVA-reactive IgG antibodies at baseline (Fig. 2a). As the pet shop mice had no known prior exposure to cOVA, the presence of such antibodies suggested the possibility that an immunostimulatory environment might drive the generation of cross-reactive immunological memory. To better characterize the reactivities, we developed an epitope display tool based on the eCPX (enhanced circularly permuted outer membrane protein OmpX) system^27^ (Extended Data Fig. 5a,b). Peptide libraries derived from cOVA and ovalbumin (OVA) orthologues from several avian species were generated for panning against antibodies. We tested the epitope display tool using monoclonal antibodies with defined reactivities by flow cytometry (Extended Data Fig. 5c,d) and by next-generation sequencing (Extended Data Fig. 5e), enriching the known epitope regions targeted by each monoclonal antibody and similar epitopes in orthologues. Sera from cOVA-immunized SPF mice enriched multiple cOVA-derived peptides, whereas cOVA-naive SPF mice showed minimal epitope enrichment (Fig. 2b). By contrast, sera from pet shop mice had diverse cOVA epitope enrichment even without prior cOVA immunization (Fig. 2b). This suggested that there may not have been a single antigen in the pet shop environment that gave rise to cOVA cross-reactivity, but rather the sheer accumulated memory repertoire reacted as a ‘stochastic ensemble’ with idiosyncratic sets of cOVA epitopes. To explore cross-reactive antigen space, albeit with reversed directionality, we screened a cOVA-reactive monoclonal antibody originally isolated from a cOVA-immunized mouse^28^ against a universal peptide epitope library^29^. We observed a remarkable breadth of reactivity: although the native cOVA epitope (LPGFGD) was detected, it was only the 99th most-enriched epitope (Extended Data Fig. 5f). Additionally, thousands of diverse epitopes gave signal above background, with clear amino acid positional preferences among the most-enriched targets (Extended Data Fig. 5g). However, several strongly enriched epitopes bore no sequence resemblance to the cOVA epitope used to select the original antibody (for example, KAASWA, ranked 325th). The impressive range of reactivities for a single monoclonal antibody lends credence to the idea that in triggering multitudinous immune responses, the pet shop environment might drive the generation of cross-reactive antibodies towards many otherwise novel antigens.

Using our epitope display system, we were also interested in characterizing the fine reactivities of additional inbred SPF strains following cOVA immunization. The targeted epitopes appeared to be strain-dependent with potential genetic and environmental contributions (Supplementary Fig. 3a,b). Unexpectedly, mouse strains with shared MHC haplotypes had divergent epitope preferences (Supplementary Fig. 3b–e). It therefore seems plausible that several layers of selection shape the constellation of antibody reactivities seen in particular strains or housing conditions. Overall, these data suggested that the presence of pre-existing cOVA-reactive antibodies in pet shop mice was dependent on the environment, whereas the fine reactivities probably depended on both the antigenic stimulus and individual genetics.

**Extended Data Fig. 6:**
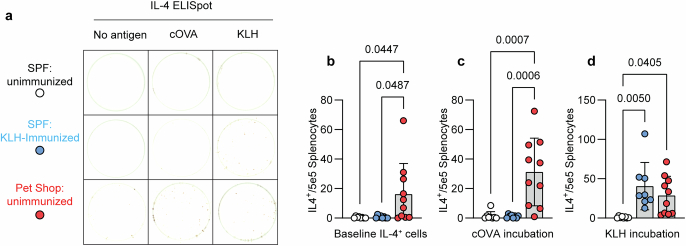
IL-4^+^ splenocytes as measured with ELISpot. (**a**) Representative ELISpot images of IL-4^+^ splenocytes from the indicated sources incubated without antigen or with cOVA or KLH. (**b-d**) quantification of IL-4^+^ spots at baseline (**b**), after incubation with cOVA (**c**), or after incubation with KLH (**d**) (n = 8 SPF/null, 8 SPF/KLH, 9 pet shop/null). Statistical significance was determined using one-way ANOVA with Tukey correction for multiple comparisons. Error bars represent SD. Data are pooled from two or more repeats of each experiment. Source data

As the formation of most IgG antibodies requires T cell help, we next investigated the T cell compartment in pet shop mice. Given the apparent promiscuity of memory T cell responses in adult humans^30^, it seemed possible that T cells in pet shop mice might not be immunologically naive to the model allergens used in sensitization experiments. To characterize T cell recognition of model antigens prior to immunization, we screened splenocytes from unimmunized pet shop and SPF mice, as well as SPF mice immunized with keyhole limpet haemocyanin (KLH), for cytokine production in response to antigen via enzyme-linked immunospot (ELISpot) assay. Whereas naive SPF mice, as expected, exhibited essentially no IFNγ production in response to cOVA or KLH, pet shop mice showed robust production of IFNγ after incubation with either antigen (Fig. 2c–f). We also tested for IL-4 and found negligible antigen-reactive type II memory cells in pet shop mice (Extended Data Fig. 6). These data suggested that pet shop mice had type I cOVA and KLH-reactive memory cells despite the mice never having encountered such antigens in their life histories. Substantiating this finding, progeny from pet shop mice born and raised in laboratory facilities produced mixed type I/type II humoral responses after adulthood sensitization with KLH in alum analogous to cOVA responses (Supplementary Fig. 4). As IFNγ is a major driver of B cell class-switching to IgG2 antibody subclasses^31^, pre-existing, antigen-reactive memory T cells producing IFNγ may explain the mixed type I/type II antibody production in pet shop mice following antigen exposure in alum. An orthogonal flow cytometry-based approach^32^ further confirmed the presence of cOVA-reactive memory CD4^+^ T cells in unimmunized pet shop mice (Fig. 2g,h and Supplementary Fig. 5). These data are consistent with findings in humans^30,33–35^ and suggest a possible adaptive immune mechanism by which allergic sensitization is temporally biased to the early-life period by virtue of limited cumulative immune experience.

## A perinatal window for allergy

**Fig. 3:**
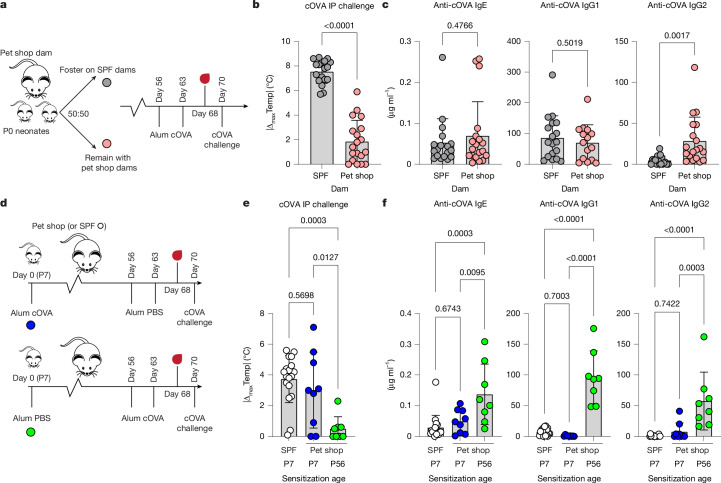
A perinatal window for allergic sensitization in pet shop mice. **a**, Fostering scheme. Pet shop neonates (postnatal day 0 (P0)) born in house were fostered by SPF dams or kept with pet shop dams. On reaching adulthood, the mice were sensitized subcutaneously with cOVA/alum, bled on day 68 and challenged intraperitoneally with cOVA on day 70. **b**,**c**, Maximal temperature decrease following cOVA challenge (**b**) and cOVA-reactive serum antibodies of the indicated isotype (**c**) (SPF dam: *n* = 18; pet shop dam: *n* = 19). **d**, Experimental scheme for perinatal (blue) or adult (green) pet shop sensitization to cOVA (with SPF controls in white). **e**,**f**, Maximal temperature decrease following cOVA challenge (**e**) and cOVA-reactive serum antibodies of the indicated isotype (**f**) (SPF P7: *n* = 18; pet shop P7: *n* = 9; pet shop P56: *n* = 8). For perinatal sensitization, three of nine pet shop mice died; for adult sensitization, none of the mice died. Two-tailed unpaired Student’s *t*-test (**b**,**c**) or one-way ANOVA with Tukey correction for multiple comparisons (**e**,**f**). Error bars represent s.d. Data are pooled from two or more repeats of each experiment. Source data

**Extended Data Fig. 7:**
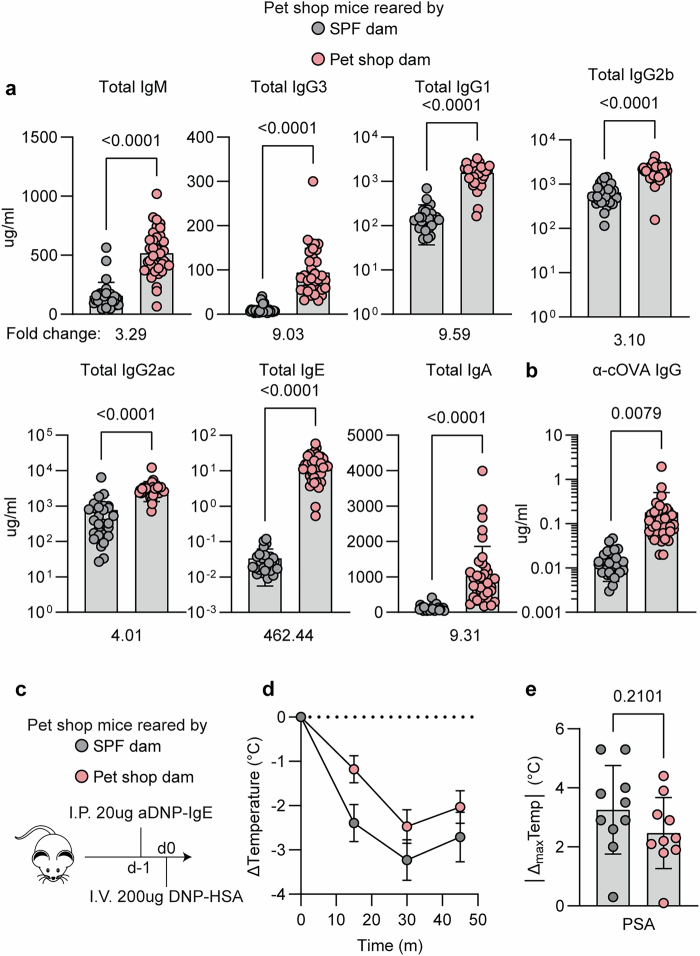
Baseline serum immunoglobulins and passive systemic anaphylaxis in fostered pet shop mice. (**a**) Baseline serum immunoglobulin levels and (**b**) baseline cOVA-reactive IgG in fostered or control pet shop mice (n = 28 SPF dam, 37 pet shop dam). (**c-e**) Passive systemic anaphylaxis scheme (**c**), temperature loss following challenge (**d**) and maximal temperature loss (**e**) in fostered or control pet shop mice (n = 10). Statistical significance was determined using two-tailed unpaired Student’s t-test. Error bars represent SD besides (**d**) (SEM). Data are pooled from two or more repeats of each experiment. Source data

**Extended Data Fig. 8:**
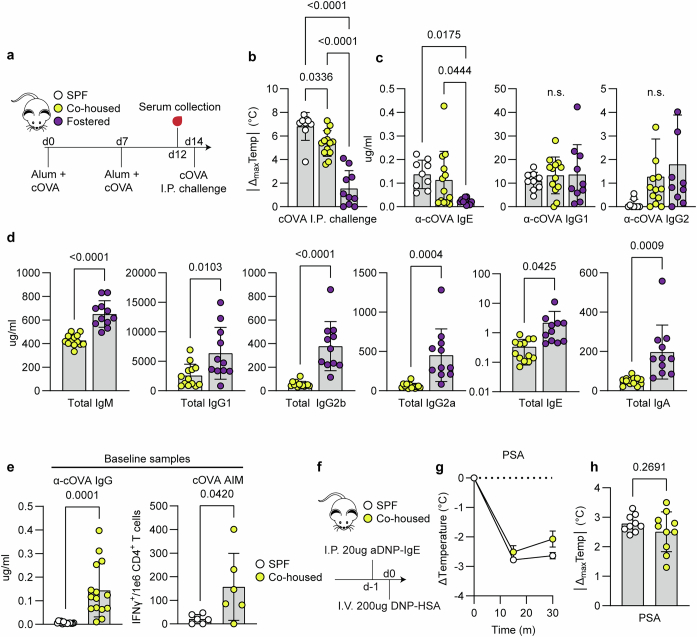
Sensitization, baseline serum immunoglobulin and passive systemic anaphylaxis in co-housed and fostered inbred mice. (**a**) Experimental scheme. SPF, co-housed progeny, and pet shop-fostered inbred mice (all BALB/cJ) were subjected to the standard sensitization protocol. Serum was collected on d12, and mice were I.P. challenged with cOVA on d14. (**b**) Maximal temperature loss following systemic cOVA challenge and (**c**) cOVA-reactive antibodies (n = 9 SPF, 13 co-housed, 10 fostered). (**d**) Baseline serum immunoglobulins (n = 13 co-housed, 11 fostered). (**e**) Baseline cOVA-reactive IgG (n = 14 SPF, 15 co-housed) and IFNγ^+^ CD4^+^ T cells (n = 6) in SPF and co-housed mice. (**f-h**) Passive systemic anaphylaxis scheme (**f**), temperature loss following challenge (**g**) and maximal temperature loss (**h**) in co-housed or control BALB/cJ mice (n = 9 SPF, 10 co-housed). Statistical significance was determined using one-way ANOVA with Tukey correction for multiple comparisons (**b,c**) or two-tailed unpaired Student’s t-test (**d,e,g**). Error bars represent SD besides (**g**) (SEM). Data are pooled from two or more repeats of each experiment. Source data

It is generally appreciated that, through an unknown mechanism, adult human immune responses to alum-based vaccines yield mixed type I/type II memory, in contrast to the strongly type II-polarized responses seen in infants after identical vaccination^36,37^. The peculiar production of antigen-reactive IgG2 antibodies (subclasses associated with type I immunity^31^) in pet shop mice following alum-adjuvanted sensitization thus resembled an adult human humoral response. To test for an environmental role in such immune deviation, and to better control for the outbred genetics of pet shop mice, we fostered pet shop neonates onto SPF dams (Fig. 3a). Fostered pet shop mice had serum immunoglobulins that were consistent with those of inbred SPF mice and were greatly reduced compared to those of unfostered littermates (Extended Data Fig. 7a). Additionally, SPF-fostered pet shop mice had no baseline cOVA-reactive IgG (Extended Data Fig. 7b). Pathogen testing indicated that we had successfully prevented infection of fostered pet shop mice with agents that were not present in our SPF colonies (Supplementary Table 2). Of note, fostered pet shop mice were highly susceptible to allergic sensitization and anaphylaxis in adulthood (Fig. 3b). Serologically, fostered pet shop mice had diminished allergen-reactive IgG2 post-sensitization while maintaining equal levels of other isotypes (Fig. 3c). We confirmed that fostered pet shop mice and littermate controls were similarly responsive to passive systemic anaphylaxis (Extended Data Fig. 7c–e). Conversely, co-housing or fostering SPF mice with pet shop cagemates or dams also skewed the humoral response away from IgE, ultimately leading to protection from anaphylaxis (Extended Data Fig. 8a–c). Co-housing or fostering in this direction did not transfer all pathogens to inbred mice, nor did it result in equally elevated total serum immunoglobulins (Extended Data Fig. 8d and Supplementary Table 3). However, co-housed inbred mice possessed both cOVA-reactive IgG and cOVA-reactive memory CD4^+^ T cells at baseline in adulthood (Extended Data Fig. 8e). Environmental effects again did not seem to alter the response to passive systemic anaphylaxis (Extended Data Fig. 8f–h). Together, our reciprocal fostering experiments suggested that the non-SPF environment was consistently altering adaptive immune responses towards an allergy-protective setpoint.

If environmental effects drive protective immune deviation, then sensitizing pet shop mice in early life, before substantial antigen or pathogen exposure, should enable the more type II-polarized immune responses detected in infant humans and adult SPF mice. Progeny of pet shop mice bred in laboratory facilities were sensitized either perinatally or as adults and systemically challenged with allergen at 11 weeks of age (Fig. 3d). Notably, only perinatally sensitized pet shop mice showed severe allergic responses, even leading to death in one-third of challenged mice (Fig. 3e). Pet shop mice sensitized early in life had type II-biased antigen-reactive humoral responses similar to those seen in inbred SPF mice sensitized at any age (Fig. 3f). By contrast, adult sensitization led to significant antigen-reactive IgG production and protection from anaphylaxis, consistent with our previous experiments (Fig. 3e,f). While maternally derived antibodies can affect adaptive immune responses in the perinatal period (Supplementary Fig. 6a–c), the similar perinatal sensitization responses of SPF and pet shop mice (Fig. 3f) indicated a limited role for maternal antibodies in these settings. Together, these results suggest that pet shop mice, similar to humans, are permissive to allergic sensitization during an early-life window, whereas in SPF mice and SPF-fostered pet shop mice, the permissive window never closes.

Given that pet shop mice transitioned from a state of allergic susceptibility perinatally to a state of protection as adults, we tested whether pet shop mice rendered allergic early in life would maintain an allergic state after further sensitizing allergen exposures in adulthood. We administered additional subcutaneous cOVA/alum doses to perinatally sensitized pet shop mice at eight weeks of age, taking serum samples before and afterwards for analysis (Supplementary Fig. 6d). Unexpectedly, additional allergen immunizations protected mice from anaphylaxis and reversed the type II-biased humoral response generated in early life, selectively increasing antigen-reactive IgG levels without altering IgE (Supplementary Fig. 6e,f). Combined, these data support a model in which immunostimulatory environments, as a function of time, drive protective mixed type I/type II immune responses to potentially sensitizing allergen exposures. Notably, we found that allergic sensitivity may not represent an indelible configuration of adaptive immunity.

## Cross-reactive memory can limit allergy

**Fig. 4:**
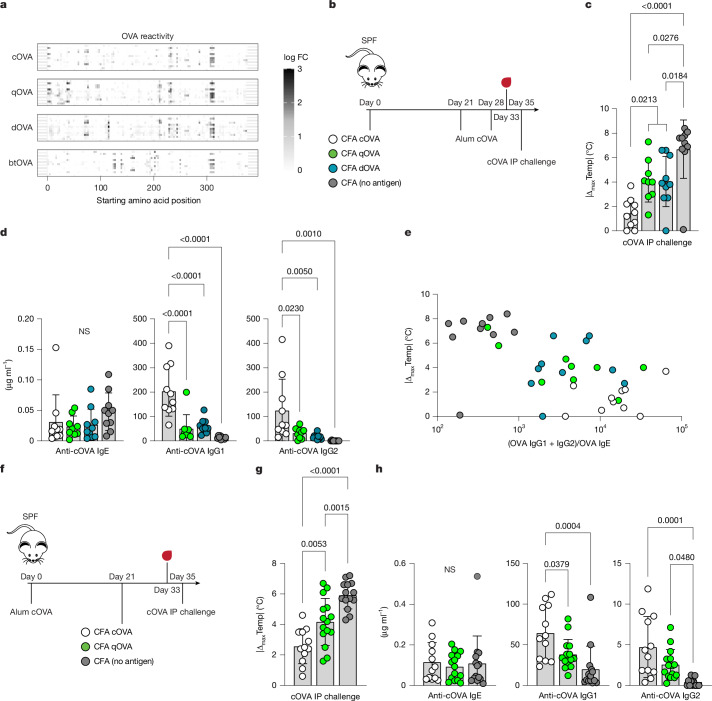
Cross-reactive type I antigen exposure protects mice from allergy. **a**, Epitope profiling of IgG reactivity against indicated OVA proteins from cOVA-sensitized SPF (C57BL/6J) mice (*n* = 12). Each row represents one sample matched across OVA proteins. OVA proteins are ordered according to sequence conservation relative to cOVA. **b**, Experimental scheme for pre-sensitization type I immune polarization. SPF mice were subcutaneously injected with CFA emulsions containing OVA orthologues or without additional antigen on day 0. Three weeks later, mice were subcutaneously injected on the contralateral flank with cOVA adsorbed to alum. cOVA/alum injections were repeated one week later, serum samples were collected after five days, and mice were challenged intraperitoneally with cOVA for measurement of anaphylaxis.0 **c**,**d**, Maximal temperature decrease following systemic cOVA challenge (**c**) and cOVA-reactive serum antibodies of the indicated isotypes (**d**) (CFA qOVA: *n* = 9; otherwise, *n* = 10). **e**, Maximal temperature decrease as a function of combined cOVA-reactive IgG concentration divided by cOVA-reactive IgE concentration. **f**, Experimental scheme for post-sensitization type I immune polarization. SPF mice were subcutaneously injected with cOVA adsorbed to alum and three weeks later were subcutaneously injected on the contralateral flank with the indicated CFA preparation. Serum samples were collected after 12 days, and mice were challenged intraperitoneally with cOVA after a further two days. **g**,**h**, Maximal core temperature decrease following systemic cOVA challenge (**g**) and cOVA-reactive serum antibodies of the indicated isotypes (**h**) (CFA cOVA: *n* = 12; CFA qOVA: *n* = 15; CFA (no antigen): *n* = 14). One-way ANOVA with Tukey correction for multiple comparisons (**c**,**d**,**g**,**h**). Error bars represent s.d. NS, not significant. Data are pooled from two or more repeats of each experiment. Source data

To assess the protective role of type I memory cells on subsequent type II antigen encounter, we used reductionist approaches in SPF mice. In the first approach, using a type I-driving *Listeria monocytogenes* infection model, we found that mice were protected from allergy in an antigen-dependent manner, although prior infection had an antigen-independent effect on anaphylaxis severity (Supplementary Fig. 7). In the second approach, we tested whether type I exposure to cross-reactive antigens would be sufficient for protection. Orthologues of cOVA were selected from a variety of avian species, including quail, duck and blue tit (qOVA, dOVA and btOVA, respectively), for which we also generated epitope display libraries. Each OVA occupies discrete intervals of antigenic space away from cOVA (91, 81 and 70% identical, respectively) and they cover the range of diversity seen in avian OVA proteins (Supplementary Fig. 8a,b). OVA proteins were purified from fresh eggs or, in the case of blue tit, produced recombinantly. Using sera from SPF mice sensitized to cOVA, we noted that the antibodies enriched epitope regions across the four OVA proteins with varying degrees of conservation (Fig. 4a). Extending this finding, cOVA-immunized SPF mouse serum reacted against crude egg white preparations across and beyond class Aves, essentially replicating a longstanding observation in the field of serology^38–41^ (Supplementary Fig. 8c,d). These data imply that exposure to a single OVA is likely to influence the adaptive immune responses to other OVA antigens.

To test the effect of heterologous OVA exposure on allergic sensitization, SPF mice were exposed to cOVA or OVA orthologues emulsified in complete Freund’s adjuvant (CFA), which drives predominantly type I immune responses, before the standard allergic sensitization protocol using cOVA (Fig. 4b). Mice that received cOVA, qOVA or dOVA were protected from subsequent sensitization with cOVA, whereas mice that received CFA alone prior to cOVA sensitization were fully anaphylactic upon challenge (Fig. 4c). Immunizations prior to sensitization did not appear to alter eventual cOVA-reactive IgE levels but did drive increased production of cOVA-reactive IgG (Fig. 4d). Protection from anaphylaxis correlated with the ratio of cOVA-reactive IgG to IgE present at time of challenge, in line with data from pet shop mice and from passive IgG transfer experiments (Fig. 4e). These results indicated that by prior exposure to an orthologue in CFA, swaths of OVA antigenic space were imprinted with type I contextual information capable of altering subsequent adaptive responses. Imprinting effects may also divert antigen-reactive B cells to suboptimal local maxima on an affinity landscape^42^. As allergic responses typically require high-affinity antibodies^43^ (Supplementary Fig. 8e) and exposure to different OVA orthologues biased the resulting antibody reactivities (Supplementary Fig. 8f,g), it was of interest to characterize antibody ‘preference’ as a proxy for affinity using sera from mice exposed to single or multiple OVA orthologues. Antibodies from mice that received heterologous immunizations preferred the first OVA orthologue encountered, consistent with the phenomenon of ‘original antigenic sin’^42^ (Supplementary Fig. 9), which, together with type I imprinting effects, suggested that prior orthologous antigen exposure may skew both the character and quality of subsequent adaptive immune responses.

The experimental results thus far warranted an investigation into the reversal of established allergic sensitivity using antigen orthologues. SPF mice were sensitized to cOVA and later exposed to cOVA or qOVA in the context of CFA (Fig. 4f). Sensitized mice treated with CFA plus cOVA or qOVA were protected from anaphylaxis compared with controls (Fig. 4g). In line with the hypothesis that non-IgE antibodies interfere with anaphylaxis in this experimental system, post-sensitization exposure to cOVA or qOVA in a type I setting resulted in a comparative increase in cOVA-reactive IgG over IgE (Fig. 4h). Combined, the reductionist experiments in SPF mice phenocopied the results of pet shop mice, suggesting that environmentally generated cross-reactive memory is a potential mechanism of protection from allergy.

## Cross-tolerance also limits allergy

**Fig. 5:**
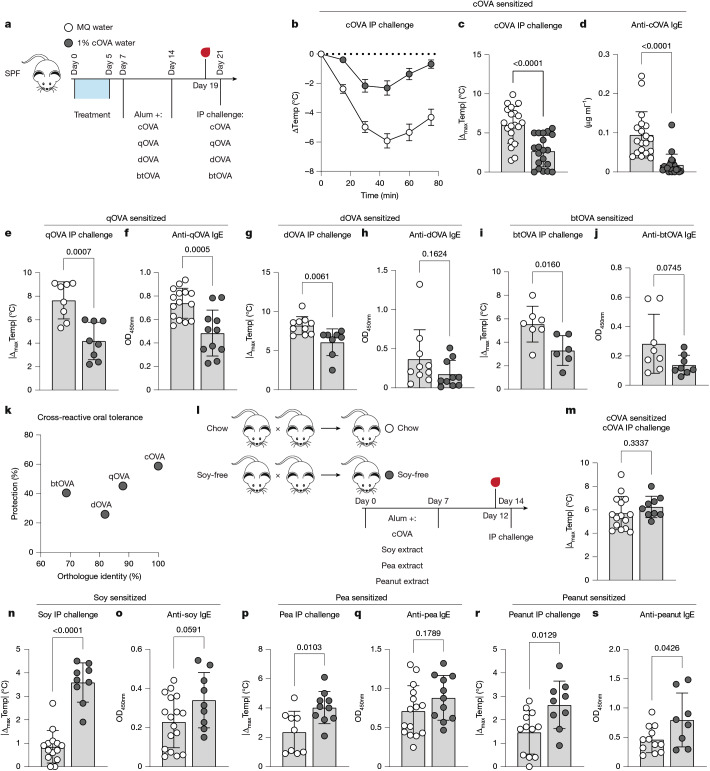
Cross-tolerance protects mice from allergy. **a**, Experimental scheme for the induction of oral tolerance by cOVA in water in SPF (BALB/cJ) mice. MQ water, Milli-Q water control. **b**, Measurement of core body temperature following systemic challenge in mice that were sensitized to and challenged intraperitoneally with cOVA. **c**,**d**, Maximal temperature decrease following systemic cOVA challenge (**c**) and cOVA-reactive serum IgE (**d**) (*n* = 20). Colour scheme as in **a**. **e**–**j**, Maximal temperature decrease (**e**,**g**,**i**) and antigen-reactive IgE (**f**,**h**,**j**) in mice sensitized to and challenged with the indicated OVA orthologue. Colour scheme as in **a**. **e**, *n* = 8. **f**, MQ: *n* = 11; cOVA: *n* = 15. **g**, MQ: *n* = 10; cOVA: *n* = 8. **h**, *n* = 10. **i**, MQ: *n* = 7; cOVA: *n* = 6. **j**, *n* = 8. **k**, Per cent protection of cOVA oral tolerance on sensitization to OVA orthologues. **l**, Experimental scheme for generating cohorts and measuring the effect of legume cross-tolerance. SPF (BALB/cJ) breeders were placed on chow or soy-free (and therefore, legume-free) diets. Progeny from each group were weaned onto matched diets and subcutaneously injected with the indicated legume extracts adsorbed to alum twice, with injections spaced one week apart. Serum samples were collected five days after the last injection, and mice were challenged intraperitoneally with matched legume extracts two days after serum collection. **m**, Maximal temperature decrease after systemic cOVA challenge (chow: *n* = 15; soy-free: *n* = 9). **n**–**s**, Maximal temperature decrease (**n**,**p**,**r**) and legume-reactive IgE (**o**,**q**,**s**) in mice sensitized to the indicated legume extract. **n**, Chow: *n* = 14; soy-free: *n* = 9. **o**, Chow: *n* = 17; soy-free: *n* = 9. **p**, Chow: *n* = 9; soy-free: *n* = 10. **q**, Chow: *n* = 15; soy=free: *n* = 11. **r**, Chow: *n* = 12; soy-free: *n* = 9. **s**, Chow: *n* = 12; soy-free: *n* = 8. Two-tailed unpaired Student’s *t*-test (**c**–**j**,**m**–**s**). Error bars represent s.d. (**c**–**j**,**m**–**s**) or s.e.m. (**b**). Data are pooled from two or more repeats of each experiment. Source data

**Extended Data Fig. 9:**
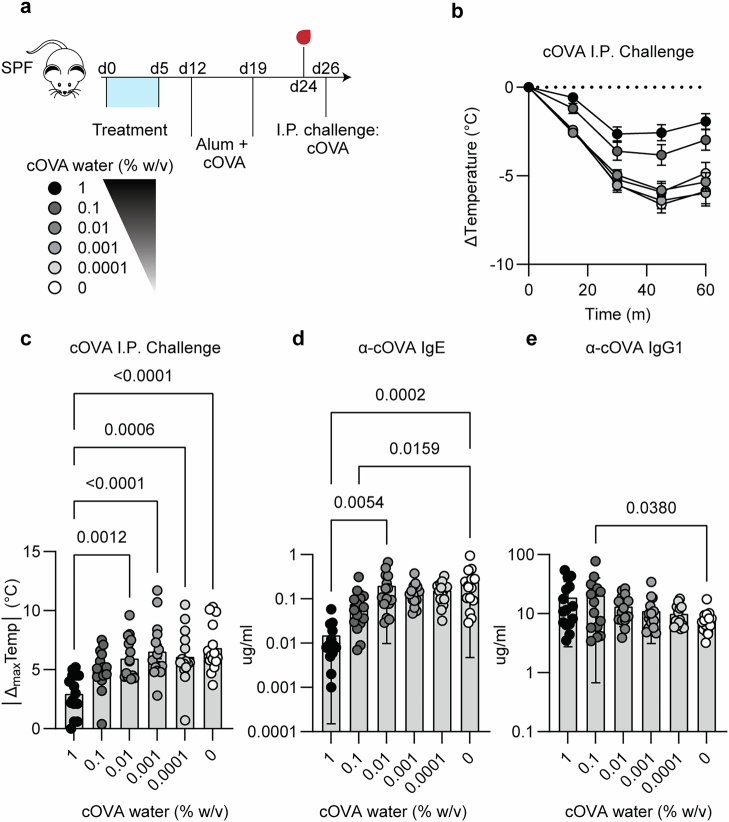
Tolerance effects as a function of antigen dose. (**a**) Experimental scheme. Mice were provided with water containing cOVA across a range of concentrations for five days. A week after water bottle removal, mice were sensitized subcutaneously with cOVA in alum. (**b**) Core body temperature following systemic challenge with cOVA. (**c**) Maximal temperature loss. (**d-e**) cOVA-reactive IgE (**d**) and IgG1 (**e**) on d24 of the experiment. n = 15 for all panels. Statistical significance was determined using one-way ANOVA with Tukey correction for multiple comparisons (**c-e**). Error bars represent SD besides (**b**) (SEM). Data are pooled from two or more repeats of each experiment. Source data

Although healthy humans have readily detectable type I memory T cells and IgG antibodies that are reactive to allergens^44–47^, a major mechanism of protection from allergy is thought to be regulatory T cell-mediated allergen tolerance. We reasoned that when administered in a tolerogenic rather than type I context, cross-reactivity between antigens should operate analogously in the suppression of allergic sensitization. In a model of oral tolerance^48^ (Fig. 5a), cOVA-tolerized mice sensitized to cOVA had reduced cOVA-reactive IgE, leading to weaker anaphylactic responses upon challenge, as expected (Fig. 5b–d). Similarly, cOVA-tolerized mice that were sensitized and challenged with various OVA orthologues were protected from full sensitization via reduced IgE, again spanning the entire identity range of the avian OVA protein family (Fig. 5e–j). There appeared to be a general correlation between orthologue similarity to cOVA and the strength of cross-tolerance (Fig. 5k). Supporting this interpretation, further testing using homologous antigens indicated that the tolerant state is quantitative in nature (Extended Data Fig. 9). These results suggest that, as with prior type I exposure, oral tolerance to a given protein imprints across a range of antigenic space. Together, these data imply that the robust type II polarization that underlies severe allergy might occur more frequently in individuals with limited prior exposure, in either type I or tolerogenic contexts, to proteins occupying overlapping antigenic space.

Administering individual antigens was useful in identifying mechanisms of cross-reactive protection from allergy, but it did not reflect natural antigen exposures, during which the immune system engages with many antigens simultaneously. Therefore, we were interested in testing the relevance of cross-reactivity in complex antigen exposures. A candidate protein source for experimentation was the legume family, which includes peanut, a common food allergen source in humans^49^. Water-soluble extracts from pea, peanut and soy contained dozens of distinct proteins, some of which had orthologous sequence similarities and some of which were unique to a given legume source (Supplementary Fig. 10). SPF breeding cages were fed commercially available mouse chow (which contains soy but no other legume) or soy-free chow devoid of any legume-derived proteins (Fig. 5l). Progeny from these breeding cages were kept on matched diets throughout the experiment (Fig. 5l). After reaching adulthood, mice on either diet were subcutaneously immunized with cOVA or one of the three legume extracts in alum and later systemically challenged with the corresponding extract. Mice on chow and soy-free diets sensitized to and challenged with cOVA were equally anaphylactic, suggesting that the diets did not generally affect allergic sensitivity to all antigens (Fig. 5m). As expected, the presence of soy in the diet provided cognate tolerance, with mice on soy-containing chow diet exhibiting protection from anaphylaxis when challenged with soy extract, compared to mice on the soy-free diet (Fig. 5n). More notably, the presence of soy in the diet also conferred protection against sensitization and anaphylaxis to the other legumes—pea and peanut (Fig. 5p,r). Mice raised on soy-free diets had subtly higher antigen-reactive IgE (Fig. 5o,q,s), in line with oral tolerance experiments using cOVA and OVA orthologues.

**Extended Data Fig. 10:**
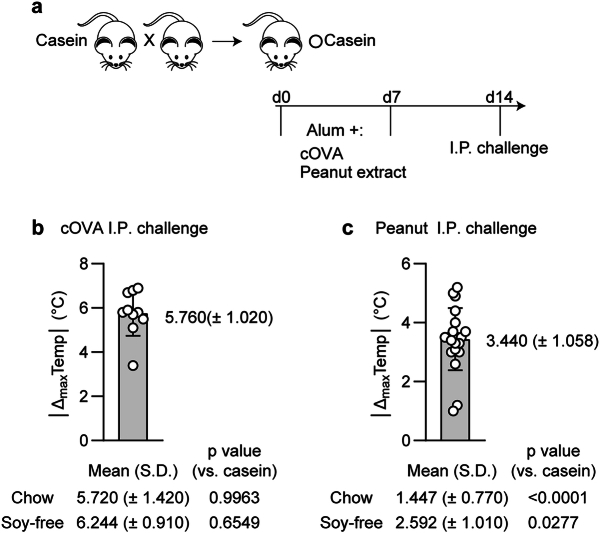
Casein-only diet allows for stronger anti-peanut responses. (**a**) Experimental scheme. Breeder pairs (BALB/cJ) were kept on casein-only diet and progeny were weaned and maintained on casein-only diet for the duration of the experiment. Upon reaching adulthood, mice were injected subcutaneously with cOVA or peanut extract and then challenged systemically. (**b**) Maximum core body temperature loss in mice after cOVA sensitization and challenge (n = 10). (**c**) Maximum core body temperature loss in mice after peanut extract sensitization and challenge (n = 20). Statistical significance was determined using one-way ANOVA with Tukey correction for multiple comparisons against regular chow and soy-free data presented in Fig. 4. Error bars represent SD. Data are pooled from two or more repeats of each experiment. Source data

Because cross-tolerance appeared to extend across an unexpectedly wide range of orthologue similarities, we considered that the remaining plant proteins in the soy-free diet, from corn and wheat, might also confer cross-tolerance during legume sensitizations. To address this possibility, SPF mice maintained on a diet with the proteinaceous fraction derived solely from bovine casein were sensitized to cOVA or peanut. Mice on the casein diet had unperturbed allergic responses to cOVA but did have heightened reactions to peanut compared to mice on regular chow or soy-free diets, suggesting that corn and/or wheat antigens may also provide some level of tolerance to legumes (Extended Data Fig. 10). These data are consistent with a recent study describing regulatory T cells that are cross-reactive to numerous plant-derived antigens^50^. Together, the effects of cross-tolerance extrapolated to complex and diverse antigen exposures, which more realistically model human antigen exposures. It is therefore likely that cross-reactive adaptive immune memory influences many responses in individuals with sufficient cumulative immune experience (Supplementary Fig. 11).

## Discussion

Allergic sensitivity appears to be a type II-imbalanced immune state and is associated with abnormal (in the evolutionary sense) modern environmental conditions. Understanding how environmental factors affect allergic sensitization was the main motivation for this study. In pet shop mice, diverse immunostimulatory exposures commensurate with a natural environmental life history broadly shifted the immune system away from a naive state, even to presumably novel antigens such as cOVA and KLH. Although our reductionist experiments suggest that this could be a dominant mechanism of protection, other alterations in pet shop mice, including chronic infection, mast cell activation and responsiveness to untested anaphylactogens, may contribute to the perceived differences in active versus passive anaphylaxis. It is noteworthy that pet shop mice appeared to be in good health despite carriage of many pathogens, including agents that are often lethal when introduced to SPF mice. We currently do not have a method of passive anaphylaxis that causes the same magnitude of systemic shock as active anaphylaxis; it is possible that pet shop mice can never achieve the same anaphylactic severity as SPF mice for reasons unrelated to adaptive immune imprinting. We envision that the adaptive immune deviation in pet shop mice is one of potentially several layers of protection against allergic sensitization and allergic anaphylaxis. It is also likely that for particular antigens, such as those derived from arthropods, cross-reactive immune memory may be imprinted towards a type II setpoint, although the effects of this with respect to allergic sensitivity are unclear^51^. Moreover, the polarization and magnitude of humoral responses in non-SPF mice could depend on other factors, including antigen dose, exposure route, choice of adjuvant and background genetics in addition to antigen-independent effects of pathogens and microbiota^52,53^. Regardless of the underlying mechanisms, several features of the immune system in pet shop mice, including mixed type I/type II responses to immunization with alum, memory reactivity to novel antigens and protection from allergic sensitization, are shared with adult humans. We must emphasize that according to our hypothetical model, environmental protection from allergy would be probabilistic, and many additional factors could contribute to allergic sensitization at any time of life.

In reductionist approaches, type I cross-reactive adaptive immune imprinting was shown to be protective against subsequent allergic sensitization. The protective effect was typified by elevated antigen-reactive IgG antibodies. In our studies, we did not determine the mechanism of IgG-mediated protection: both allergen sequestration and inhibitory Fc receptor signalling have been suggested to be involved^24–26^. Unexpectedly, type I antigen or orthologue exposure was also protective in previously sensitized mice. Given recent findings related to antigen imprinting and epitope masking^54,55^, which may operate through multiple mechanisms^56^, tracking the durability of post-sensitization protection using both cognate and orthologous antigens will clarify the therapeutic potential of such approaches.

Although cross-reactive tolerance also protected mice from allergic sensitization, the underlying protective mechanisms were reflected in diminution, rather than type I-deviation, of the humoral response. We note that the models of oral tolerance used here could have a robust and potentially exaggerated tolerogenic effect, and in general, translating oral tolerance findings to the clinic has led to less impressive results. We found that tolerance, even to cognate antigen, has a quantitative aspect and an apparent effect threshold. We speculate that the antigen doses used in most of the mouse tolerance experiments presented here, when considered in relation to organismal mass, exceed those used in humans. These data thus show only that cross-reactive tolerance is formally possible. Nonetheless, we consistently observed that type I inflammatory or tolerogenic exposure to a particular antigen conferred protection against allergic sensitization to a range of antigenically related proteins. Overall, given the numerous immunological parallels between pet shop mice and humans observed by us and by others, these findings may provide at least a partial explanation for the rise of allergic disease over the last century.

## Methods

### Mice

Pet shop mice were purchased from Komodo Reptile. Mice of the following strains were purchased from The Jackson Laboratory: 129S1/SvImJ (strain 002448), A/J (000646), BALB/cJ (000651), CAST/EiJ (000928), C3H/HeJ (000659), C57BL/6J (000664), DBA/1J (000670), FVB/NJ (001800), PERC/EiJ (001307), PWK/PhJ (003715) SJL/J (000686) and WSB/EiJ (001145). C57BL/6J or BALB/cJ mice were used in SPF experiments as indicated. Except where noted, all mice were kept on standard chow diet (Envigo/Inotiv 2018S). In experiments using soy-free diet (Envigo/Inotiv 2020SX), breeder pairs were placed onto soy-free diet and progeny to be used for experiments were weaned onto, and maintained, on a soy-free diet. For experiments using casein diet, breeder pairs and progeny were maintained on a custom 18% casein protein diet (Envigo/Inotiv TD170404). Mice were 8–12 weeks of age at the initiation of experimental procedures except where noted. Experimental cohorts bred in house comprised female and male mice; female mice were used in all other experiments. Cages of mice were randomized across conditions and sample sizes were not determined a priori. Workers were not blinded to treatment conditions. All protocols were reviewed, approved, and conducted under the institutional regulation of Yale University’s Institutional Animal Care and Use Committee.

### Pathogen testing and cytokine measurement

Oral swabs, fur swabs and faecal samples were submitted to IDEXX BioAnalytics and screened by PCR for the presence of common mouse pathogens. Serum cytokine measurement was performed by Eve Technologies.

### 16s rRNA sequencing and analysis

Immediately upon arrival at our facilities, pet shop faecal samples were collected in sterile Eppendorf tubes. DNA was isolated (Qiagen 12855) followed by amplification of the 16S rRNA gene V4 region by PCR using a dual index multiplexing strategy as previously described^57^. Successful amplification was confirmed by gel electrophoresis; amplicons were then pooled and gel-extracted. Library quantification was conducted on gel-extracted fraction (KAPA Biosystems KK4835; Applied Biosystems QuantStudio 6 Flex instrument). Paired-end sequencing (2 × 250) was performed on an Illumina Miseq using a 500-cycle V2 reagent kit (Illumina MS-102-2003).

### Co-housing and fostering

To establish a colony of inbred mice bearing pathogens, adults were co-housed with pet shop mice for two weeks, then separated into breeding cages. Progeny from these breeding pairs were used as experimental animals. For studies on baseline antibody reactivities and T cell memory, adult inbred SPF mice were co-housed with pet shop mice for 60 days and directly tested. For foster experiments, P1 neonatal inbred mice from SPF breeders were fostered onto pet shop dams and remained with the dam until weaning.

### Allergic sensitization and anaphylaxis measurement

For subcutaneous (skin) sensitization, 5 µg of low-endotoxin cOVA (Biovendor 321001) adsorbed to ~500 µg (50 µl) aluminium hydroxide gel (alum) (Invivogen vac-alu-250) with PBS to a final volume of 100 µl was injected subcutaneously in the flank near the inguinal lymph node using an insulin syringe (BD 329410). In some experiments, KLH (Sigma H7017) was used as antigen. For intranasal (lung) sensitization, 20 µg of low-endotoxin cOVA and 20 µg of papain (ThermoFisher Scientific 416760100) with PBS to a final volume of 40 µl was administered into the nasal passage of anaesthetized mice. For intragastric (intestinal) sensitization, 50 mg of cOVA grade V (Sigma A5503) and 10 µg of cholera toxin (Sigma 227036) in 200 µl bicarbonate buffer was administered by gavage. Injections or administrations were repeated seven days later, and serum was collected after an additional five days. Two days after serum collection, mice were challenged intraperitoneally with 75 µg cOVA grade V (Sigma A5503) in PBS to a final volume of 200 µl and core body temperature was measured every 15 min using a rectal thermometer (Physitemp TH-5) over the course of 75 min. Perinatal sensitizations were performed with 5 µg cOVA adsorbed to ~250 µg alum in PBS to a final volume of 50 µl injected subcutaneously in the flank.

### Pre and post-sensitization CFA immunization

Mice received subcutaneous flank injections of 100 µl containing 5 µg antigen emulsified in a 1:1 mixture of PBS and CFA (Sigma F5881). In CFA pre-treatment experiments, mice were immunized with CFA plus antigen 21 days prior to standard cOVA sensitization. For CFA post-sensitization experiments, mice were immunized with CFA plus antigen 21 days after a single 5 µg dose of cOVA in alum. All CFA plus antigen injections were administered on the contralateral flank from the cOVA plus alum injection site.

### Legume extract preparation and legume sensitization

Legume extracts were obtained by dissolving purified proteins from pea (Greer F167), peanut (Greer F171) or soybean (Greer F209) in PBS and incubated overnight at 4 °C with rotation and centrifuged at 3,214*g* for 30 min at 4 °C to remove insoluble matter. The resulting supernatant was filtered through 40-µm mesh filters (Fisher Scientific 22-363-547). Protein content of the resulting extracts was measured by BCA (Pierce 23225).

Mice received subcutaneous flank injections of 10 µg legume extract adsorbed to 500 µg alum with PBS to a final volume of 100 µl. Injections were repeated one week later. Serum was collected after an additional five days. Two days after serum collection, mice were challenged intraperitoneally with 250 µg legume extract in PBS to a final volume of 200 µl and monitored for core body temperature every 15 min over the course of 75 min.

### OVA purification and recombinant expression

Egg whites were obtained from fresh eggs purchased from local grocery stores. Egg whites were fractionated using aqueous biphasic separation procedures according to a published protocol^58^. The top phase was collected, diluted 1:5 with ice-cold PBS and incubated overnight at 4 °C. The following day, samples were centrifuged at 20,000*g* for 30 min at 4 °C, and the supernatant collected for analysis. For btOVA, no eggs were readily available for OVA purification. The coding sequence of btOVA including a C-terminal His tag was synthesized (Twist Biosciences) and expressed in Expi293F cells (ThermoFisher Scientific A14527) following the manufacturer’s specifications. The cell line was not further authenticated nor tested for mycoplasma contamination. btOVA was purified using Ni-NTA agarose beads (Qiagen 30210) and buffer exchanged into PBS on 3K protein concentrators (ThermoFisher Scientific 88512). OVA purity was assessed by gel densitometry using Mini-PROTEAN TGX stain-free gels (Bio-Rad 4568086) on a Bio-Rad Chemidoc MP imaging system.

### Passive systemic anaphylaxis

Mice received intraperitoneal injections of 20 µg of anti-DNP IgE (Sigma D8406) in PBS to a volume of 200 µl. One day later, mice received intravenous injections of 200 µg DNP–HSA (Biosearch Technologies D-5059) in PBS to a volume of 200 µl and monitored for core body temperature every 15 min over the course of 75 min.

### Anaphylaxis with compound 48/80, histamine and platelet activating factor

Mice received intraperitoneal injections of 4 mg kg^−1^ Compound 48/80 (Sigma 2313), 20 µmol histamine dihydrochloride (Sigma) or 100 µg kg^−1^ platelet activating factor-16 (Sigma) all in PBS in final injection volumes of 200 µl. Core body temperature was measured every 15 min over the course of 75 min.

### Oral tolerance model

Oral tolerance was established similar to a published protocol^48^. Mice were provided control Milli-Q water or Milli-Q water with 1% w/v cOVA grade III (Sigma A5378) ad libitum for 5 days with water replaced every other day. Mice were returned to facility water for two days, then sensitized as described.

### Listeria infection

Mice were infected intravenously with 5 × 10^4^ colony-forming units of *L. monocytogenes* strain 10403s or *L. monocytogenes* transgenically expressing cOVA^59^. Three weeks later, mice were subjected to a standard sensitization protocol followed by intraperitoneal cOVA challenge and measurement of anaphylaxis.

### ELISA

Nunc Maxisorp plates (ThermoFisher Scientific 442404) were coated with 100 µl per well of antigen or capture antibodies in coating buffer (Sigma C3041) either for 1 h at room temperature or overnight at 4 °C. Plates were washed three times with PBS/0.05% Tween-20 (PBS-T). Plates were blocked with 200 µl per well of blocking buffer (PBS-T + 1% w/v non-fat milk powder) for 1 h at room temperature or overnight at 4 °C. Standards and samples were diluted in blocking buffer and 100 µl per well was added in duplicates, followed by an incubation of 1 h at room temperature. Plates were washed six times with PBS-T, and 100 µl per well of blocking buffer with detection antibodies was added and incubated for 1 h at room temperature. Plates were again washed six times with PBS-T and developed using TMB (BD and reactions stopped by 0.18 M H_2_SO_4_). Absorbance was read on a Spectramax plate reader (Molecular Devices SpectraMax M5). Antibodies used for capture, detection and standards are listed in Supplementary Table 4.

### Blocking ELISA and antibody preference index

Sera were normalized to 10 ng ml^−1^ for anti-cOVA IgG1 or IgG2, respectively, by dilution in ELISA blocking buffer, and 50 µl per well was added to antigen-coated Nunc Maxisorp plates pre-loaded with 50 µl per well of ELISA blocking buffer with or without 200 µg ml^−1^ soluble antigen. From this point, plates were treated as described above. Antibody preference index was calculated by taking the percentage of OD_450 nm_ signal retained in cOVA-coated plates blocked with qOVA minus the percentage of OD_450 nm_ signal retained in qOVA-coated plates blocked with cOVA. A maximum value of 1 indicated complete preference for cOVA, and a minimum value of −1 indicated complete preference for qOVA.

### ELISpot

ELISpot plates (Millipore) were coated with 100 µl per well anti-IFNγ or anti-IL-4 (BD 551881,551878) at a concentration of 5 µg ml^−1^ in sterile PBS overnight at 4 °C. The following day, plates were washed three times with 200 µl PBS (5-min incubations) and twice with PBS-T. Plates were blocked with 100 µl complete RPMI medium (+10% fetal calf serum, 25 mM HEPES, 2mM l-glutamine, 1× MEM non-essential amino acids, 1× penicillin/streptomycin, 1 mM sodium pyruvate) for 2 h at 37 °C. Prior to cell addition, medium was removed and 50 µl of complete RPMI + antigen (160 µg ml^−1^) was added. Spleens were minced and dissociated in RPMI + 0.1 mg ml^−1^ DNAse I (Sigma DN25) + 0.25 mg ml^−1^ Liberase TL (Roche 05401020001) for 30 min at 37 °C with agitation. Following dissociation, cells were passed through 40-µm filters, spun at 800*g* for 5 min at 4 °C and resuspended in red blood cell lysis buffer (Biolegend 420302) according to the manufacturer’s recommended protocol. Following red blood cell lysis, 45 ml of ice-cold PBS was added, and cells were spun at 800*g* for 5 min and resuspended in 5 ml ice-cold complete RPMI. Cells were quantified on a haemocytometer and adjusted with complete RPMI to a concentration of 1 × 10^7^ cells per ml. From this, 50 µl of cells were added to ELISpot plates already containing complete RPMI with or without antigen. Plates were incubated ~18 h at 37 °C with 90% humidity and 5% CO_2_. Following incubation, cells were tapped out of plates and wells were rinsed twice with 200 µl deionized H_2_O. Biotinylated anti-IFNγ and anti-IL-4 (BD 551881,551878) were added at a concentration of 2 µg ml^−1^ in PBS at a volume of 100 µl per well. Plates were incubated for 2 h at room temperature, then washed three times with PBS-T. One hundred microlitres per well of Streptavidin–HRP (BD 557630) in PBS was added and plates were incubated for 1 h at room temperature. Spots were developed using AEC (Sigma AEC101), and the reactions were stopped by thoroughly rinsing plates with deionized H_2_O. Spots were counted using an ELISpot reader (Autoimmun Diagnostika).

### Flow cytometry

Identification of antigen-reactive CD4^+^ T cells was performed as described^33^. Splenic single cell suspensions prepared as described above were incubated at 1 × 10^7^ cells per ml in 24-well plates (Falcon 353047) with complete RPMI with or without 100 µg ml^−1^ antigen in incubators kept at 37 °C with 5% CO_2_ and 95% relative humidity. After 6 h of incubation, Brefeldin A (Sigma B7651) was added to wells followed by a further 12 h of incubation. Cells were removed from wells and incubated with aCD16/CD32 (Biolegend 101320). Surface staining was performed simultaneously with Zombie UV (Biolegend). After surface staining, cells were washed and fixed (BD 554714) according to the manufacturer’s specifications and stained for intracellular antigens. Cells were analysed on a BD Symphony flow cytometer. Data were analysed using FlowJo (Tree Star). All antibodies are listed in Supplementary Table 5.

For bacterial flow cytometry, samples were prepared as described for peptide enrichment. Following incubation with serum or monoclonal antibody, samples were stained with FITC-conjugated polyclonal goat anti-mouse IgG (Southern Biotech 103302) in PBS-T and analysed on a BD Symphony or BD LSRII flow cytometer.

### Bacterial peptide display cloning

The pB33eCPX plasmid^27^ (Addgene) was modified first by inserting an AfeI restriction site after the signal sequence embedded in a longer Gly-Ser linker. AfeI was chosen because the sequence encodes Ser-Ala, allowing for flexible display of the intervening peptide of interest. Separately, fluorescent proteins under the control of the constitutive Anderson promoter Bba_J23100 (http://parts.igem.org/) were inserted in a SalI-digested plasmid in antisense orientation relative to the peptide scaffold. Oligonucleotide pools (Integrated DNA Technologies) containing *Escherichia coli* sequence-optimized 36 bp segments of reverse translated antigen proteins plus 10 bp plasmid overhangs were amplified for a final 25 bp of plasmid homology on either side. Amplified fragments were HiFi-assembled (New England Biolabs) into AfeI-digested plasmid en masse and transformed into NEB 5-alpha chemically competent *E. coli*. Transformants were bulk cultured in lysogeny broth (LB) containing 25 µg ml^−1^ chloramphenicol. A depiction of the eCPX protein with peptide (Extended Data Fig. 5b) is based on a crystal structure of OmpX^60^ (Protein Data Bank: 1QJ8) and was produced in part using PyMol (v.2.5, Schrödinger).

### Peptide display enrichment

Peptide display enrichment was performed as described^29^ with several modifications. Bacteria containing peptide libraries were grown overnight (37 °C, 250 rpm) and diluted the following day using fresh pre-warmed LB + chloramphenicol 1:6. Upon reaching an OD of 0.6, cultures were induced for scaffold/peptide expression with 0.4% w/v l-arabinose. Induced cultures were grown for 1 h (37 °C, 250 rpm), pelleted and resuspended in PBS-T.

Serum samples were heat inactivated (56 °C, 15 min) and blocked with bacteria expressing empty eCPX scaffold in PBS-T for 1 h (4 °C, 800 rpm) in deep well plates on a Thermomixer C (Eppendorf 5382000023). Blocking bacteria were pelleted at 3,900*g* for 10 min, and supernatant was collected into a fresh deep-well plate. Pooled bacterial peptide libraries were added to wells and incubated for 1 h (4 °C, 800 rpm). Samples were washed 5 times with 1 ml PBS-T, then resuspended in 500 µl PBS containing 10 µl per well magnetic Protein G beads (NEB S1430S). Samples were incubated for 1 h (4 °C, 800 rpm), washed 5 times with 1 ml PBS between bead pull-down on a magnetic plate (Alpaqua A000400). Following final wash, bacteria were resuspend in 1 ml fresh LB + chloramphenicol + 0.2%w/v L-glucose. Bacteria were grown overnight in deep well plates (37 °C, 800 rpm). For monoclonal antibody enrichments, mouse anti-cOVA IgG1 (mAb1) (Chondrex 7094) and mouse anti-cOVA IgG2b (mAb2) (Chondrex 7096), each with defined linear epitopes on cOVA, were used. Universal peptide epitope screening and analysis were performed using mAb1 by Serimmune.

### Next generation sequencing

Plasmids from overnight cultures were isolated (Qiagen 27291) on a vacuum manifold (Qiagen 19504) and used as a template for PCR. First, amplicons containing peptide coding sequence were generated with primers F (5′-TCGTCGGCAGCGT CAGATGTGTATAAGAGACAGBHH VBCTTCCGTAGCTGGCCAGTCT-3′) and R (5′-GTCTCGTGGGCTCGGA GATGTGTATAAGAGACAGTGC CCAGACTGCCCTCC-3′). Ambiguous bases BHHVB were included in the F primer as the first five to be read during sequencing in order to avoid miscalling of clusters. Amplicons were purified with Sera-Mag SpeedBead carboxylate-modified magnetic particles (Cytiva 45152105050250) in crowding buffer as described^61^. Purified products were further amplified using indexing primers containing 8-nt indices allowing for 96 unique index pairs, purified, and quantified on a Nanodrop 8000 and with Qubit high-sensitivity assay (Invitrogen Q32851). Samples were pooled and PhiX (Illumina FC-110-3001) was added to comprise 30% of the total DNA content. Pooled samples were single-end sequenced in an Illumina P1 100 cycle cartridge (Illumina 20074933) on an Illumina Nextseq2000. De-multiplexed reads were trimmed^62^ and analysed using the Phippery suite of tools^63^. Enrichment data are displayed as log FC versus serum-free controls. Negative log FC values were set to zero for visualization. Peptides for which any bead control had zero reads were excluded from analysis.

### Statistical analysis

All statistical analyses were conducted using GraphPad Prism 9.0 (GraphPad Software).

### Reporting summary

Further information on research design is available in the Nature Portfolio Reporting Summary linked to this article.

## Online content

Any methods, additional references, Nature Portfolio reporting summaries, source data, extended data, supplementary information, acknowledgements, peer review information; details of author contributions and competing interests; and statements of data and code availability are available at 10.1038/s41586-025-10001-5.

## Supplementary information

Supplementary Information Supplementary Figs. 1–11 and Supplementary Tables 1–5.

Reporting Summary

Source Data for Supplementary Figs. 2–4 and 6–9.

## Source data

Source Data Fig. 1

Source Data Fig. 2

Source Data Fig. 3

Source Data Fig. 4

Source Data Fig. 5

Source Data Extended Data Fig. 1

Source Data Extended Data Fig. 2

Source Data Extended Data Fig. 3

Source Data Extended Data Fig. 4

Source Data Extended Data Fig. 5

Source Data Extended Data Fig. 6

Source Data Extended Data Fig. 7

Source Data Extended Data Fig. 8

Source Data Extended Data Fig. 9

Source Data Extended Data Fig. 10

## Data Availability

All data are available in the main text or the supplementary materials.
