# Author Correction: Environmentally driven immune imprinting protects against allergy

**DOI:** 10.1038/s41586-026-10236-w

**Published:** 2026-02-06

**Authors:** S. Erickson, B. Lauring, J. Cullen, R. Medzhitov

**Affiliations:** 1 Department of Immunobiology, Yale University School of Medicine, New Haven, CT USA; 2 Tananbaum Center for Theoretical and Analytical Human Biology, Yale University School of Medicine, New Haven, CT USA; 3 Howard Hughes Medical Institute, Chevy Chase, MD USA

**Keywords:** Immunology, Adaptive immunity

Correction to: *Nature* 10.1038/s41586-025-10001-5 Published online 28 January 2026

In the version of this article initially published, the images presented as Extended Data Figs. 8, 9 were interchanged. The correct Extended Data Figs. 8 (panels a–h) and 9 (panels a–e) are now updated in the HTML and PDF versions of the article.

