# Supplementary material for: Environmentally driven immune imprinting protects against allergy
Source: Nature. 2026 Jan 28;650(8103):987–96. doi: 10.1038/s41586-025-10001-5 (PMC12935535; doi:10.1038/s41586-025-10001-5)
Supplement: Supplementary file 1 — Supplementary Figs. 1–11 and Supplementary Tables 1–5. [file 41586_2025_10001_MOESM1_ESM.pdf]

nature portfolio

https://doi.org/10.1038/s41586-025-10001-5

Supplementary information

Environmentally driven immune imprinting protects against allergy

In the format provided by the authors and unedited

Supplementary Information for:

Environmentally driven immune imprinting protects against allergy

S. Erickson[1], B. Lauring[1], J. Cullen[1], R. Medzhitov[1,2,3*]

[1]Department of Immunobiology, Yale University School of Medicine, New Haven, Connecticut 06520, USA.

[2]Tananbaum Center for Theoretical and Analytical Human Biology, Yale University School of Medicine, New Haven, CT 06520, USA

[3]Howard Hughes Medical Institute

*Corresponding author. Email: ruslan.medzhitov@yale.edu

Contents:

I. Supplemental Figures
II. Supplemental Tables

Supplemental Figure List:

Supplemental Figure 1. Uncropped protein gel image.

Supplemental Figure 2. Fecal bacterial composition in SPF and pet shop mice.

Supplemental Figure 3. Epitope profiling of additional inbred SPF strains and validation of epitope targets by bacterial flow cytometry.

Supplemental Figure 4. Pet shop mice bred in-house mount mixed Type I/Type II humoral responses to alum-adjuvanted antigen exposures.

Supplemental Figure 5. Flow cytometry gating strategy for analysis of antigen-reactive CD4+ T cells.

Supplemental Figure 6. Early life sensitization in pet shop mice and SPF mice born to immune dams.

Supplemental Figure 7. Infection with cOVA-transgenic Listeria monocytogenes suppresses subsequent cOVA allergic sensitivity.

Supplemental Figure 8. Ovalbumin purification and serological cross-reactivity.

Supplemental Figure 9. Antibody binding preferences are established by primary immunization.

Supplemental Figure 10. Preparation of legume extracts and antigen correspondence between soy and pea or peanut

Supplemental Figure 11. Environmentally driven immune imprinting protects against allergy.

Supplemental Table List:

Supplemental Table 1. Pathogens detected in pet shop mice upon arrival at laboratory facilities.

Supplemental Table 2. Pathogens detected in SPF-fostered pet shop mice

Supplemental Table 3. Pathogens detected in pet shop-fostered and co-housed inbred mice

Supplemental Table 4. Antibodies used for ELISA

Supplemental Table 5. Antibodies used for flow cytometry

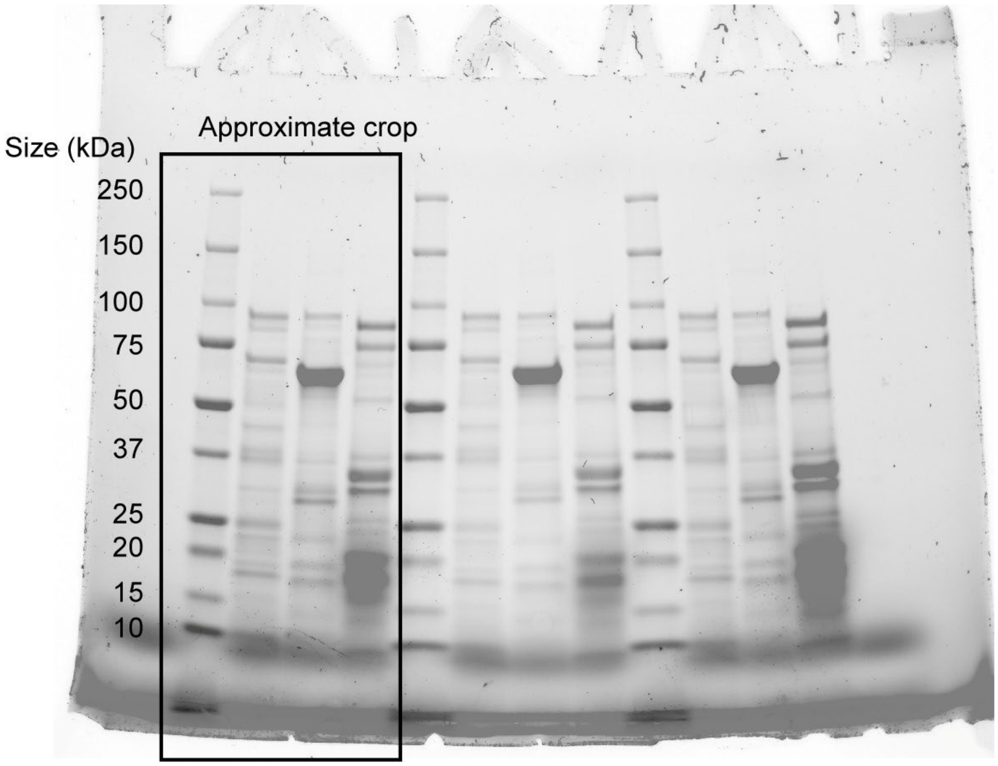

Supplemental Figure 1. Uncropped protein gel image.

Stain-free gel image of legume extracts used for experiments in Figure 5, shown cropped in Supplemental Figure 10.

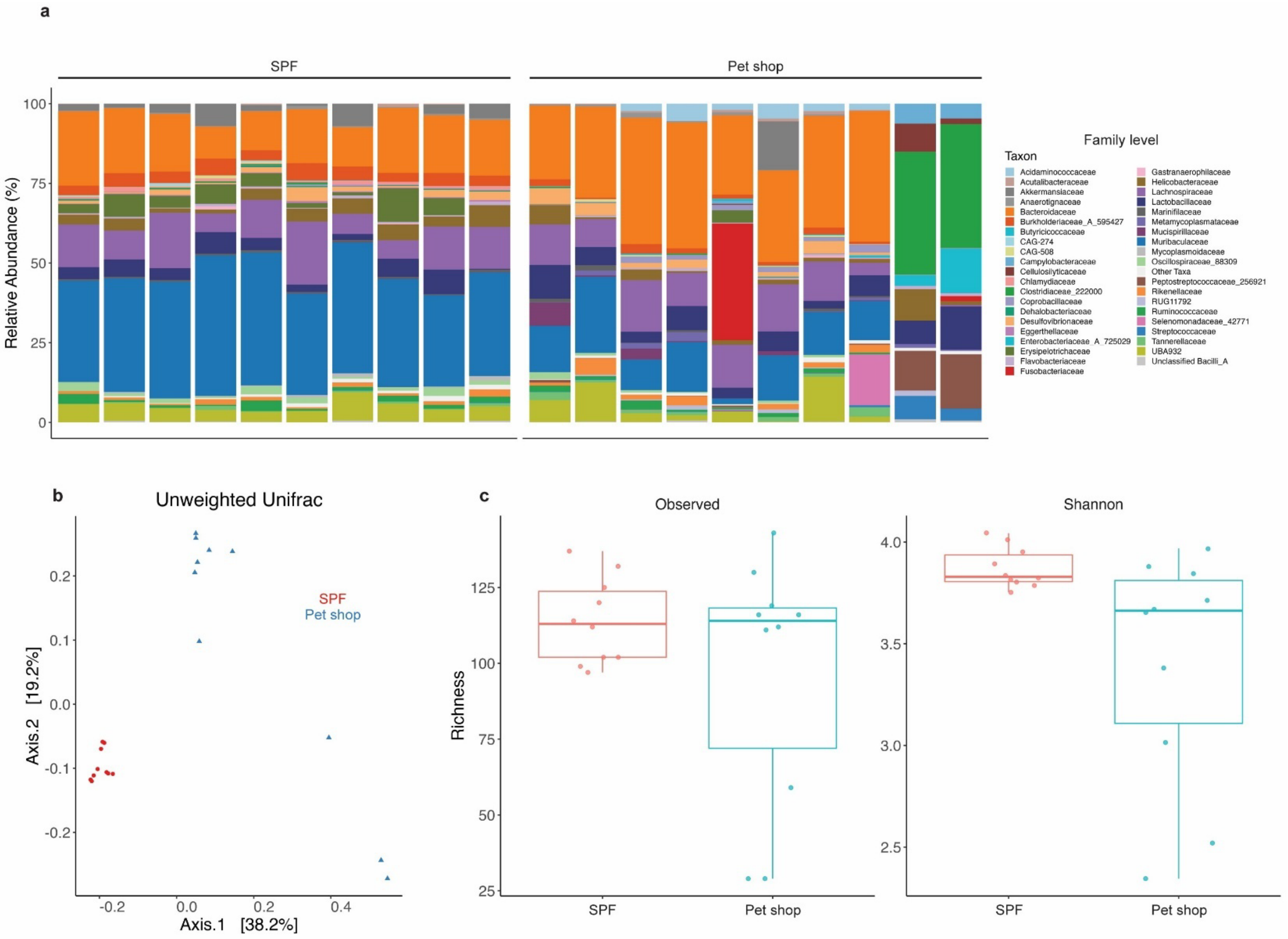

Supplemental Figure 2. Fecal bacterial composition in SPF and pet shop mice.

(**a**) Family level 16s rRNA sequencing of fecal samples from SPF and pet shop mice. (**b**) Unweighted Unifrac measurement. (**c**) Alpha diversity of fecal bacterial samples. n = 10.

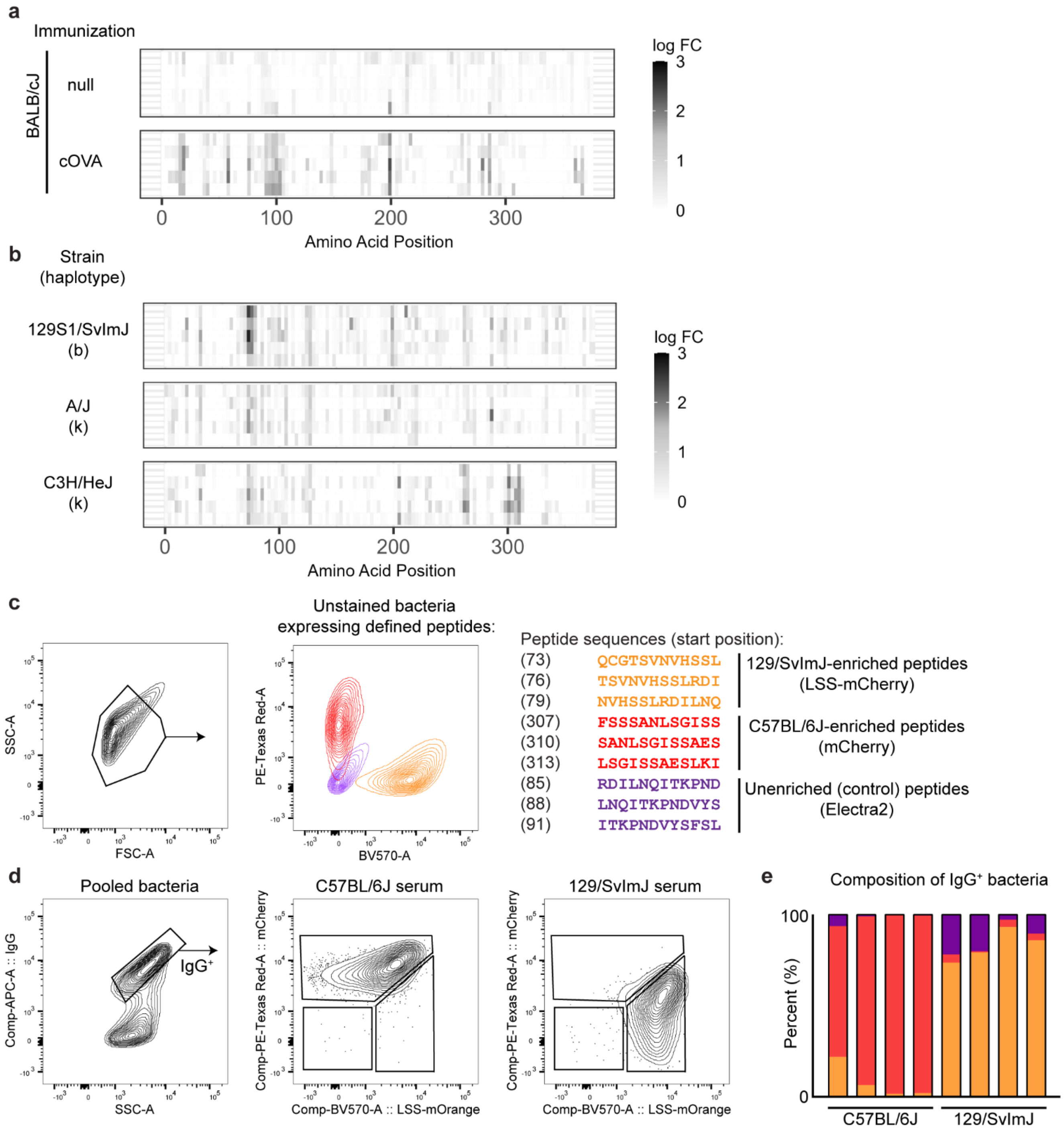

Supplemental Figure 3. Epitope profiling of additional inbred SPF strains and validation of epitope targets by bacterial flow cytometry.

(**a**) Epitope profiling of IgG reactivity against cOVA by SPF BALB/cJ mice before and after sensitization (n = 5). (**b**) Epitope profiling of IgG reactivity against cOVA by the indicated strains after sensitization (n = 5). (**c-d**) flow cytometry of bacteria expressing defined surface-bound peptides derived from cOVA discernable by flow cytometry (**c**) and following incubation with serum samples from the indicated strains after sensitization (**d**). (**e**) Composition of IgG^+ bacteria after serum incubation (n = 4 per strain).

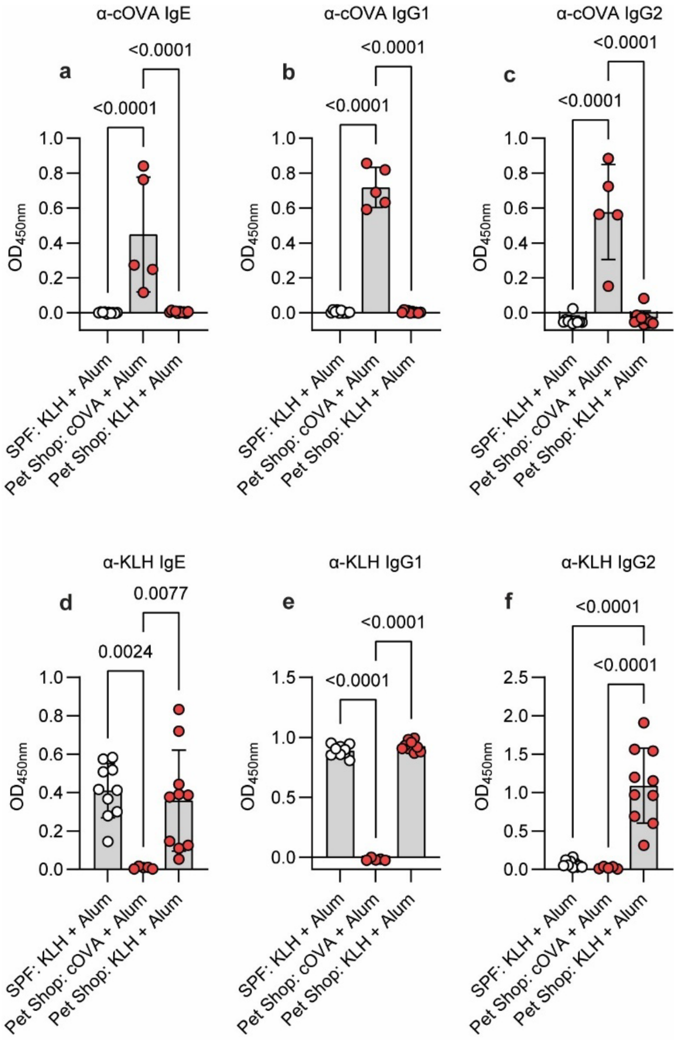

Supplemental Figure 4. Pet shop mice bred in-house mount mixed Type I/Type II humoral responses to alum-adjuvanted antigen exposures.

(**a-f**) Analysis of serum samples diluted 1:10,000 for cOVA-reactive antibodies (**a-c**) or KLH-reactive antibodies (**d-f**) from SPF (BALB/cJ) and laboratory-bred pet shop mice following skin sensitization with the indicated antigen (n = 10 SPF/KLH, 10 pet shop/KLH, 5 pet shop/cOVA). Statistical significance was determined using one-way ANOVA with Tukey correction for multiple comparisons. Error bars represent SD.

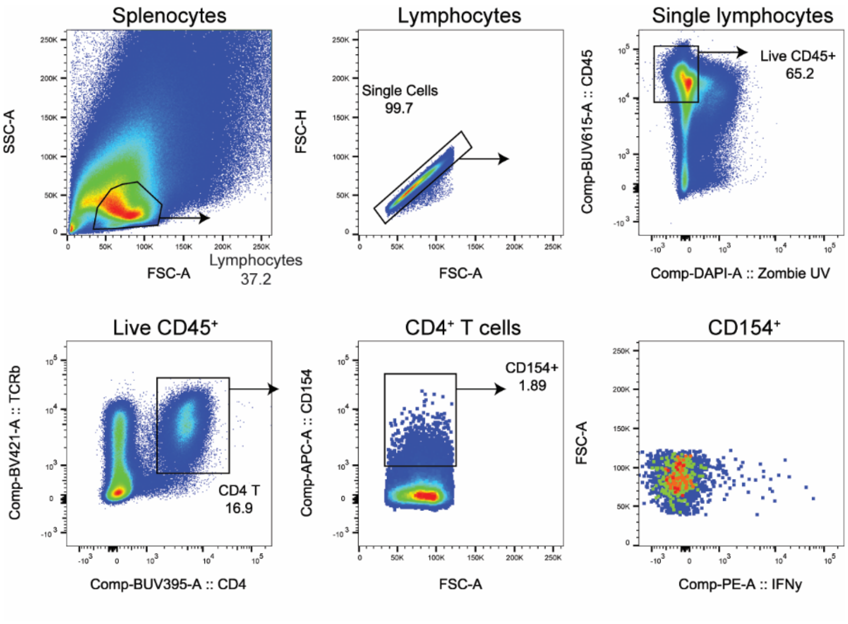

Supplemental Figure 5. Flow cytometry gating strategy for analysis of antigen-reactive CD4^{+} T cells.

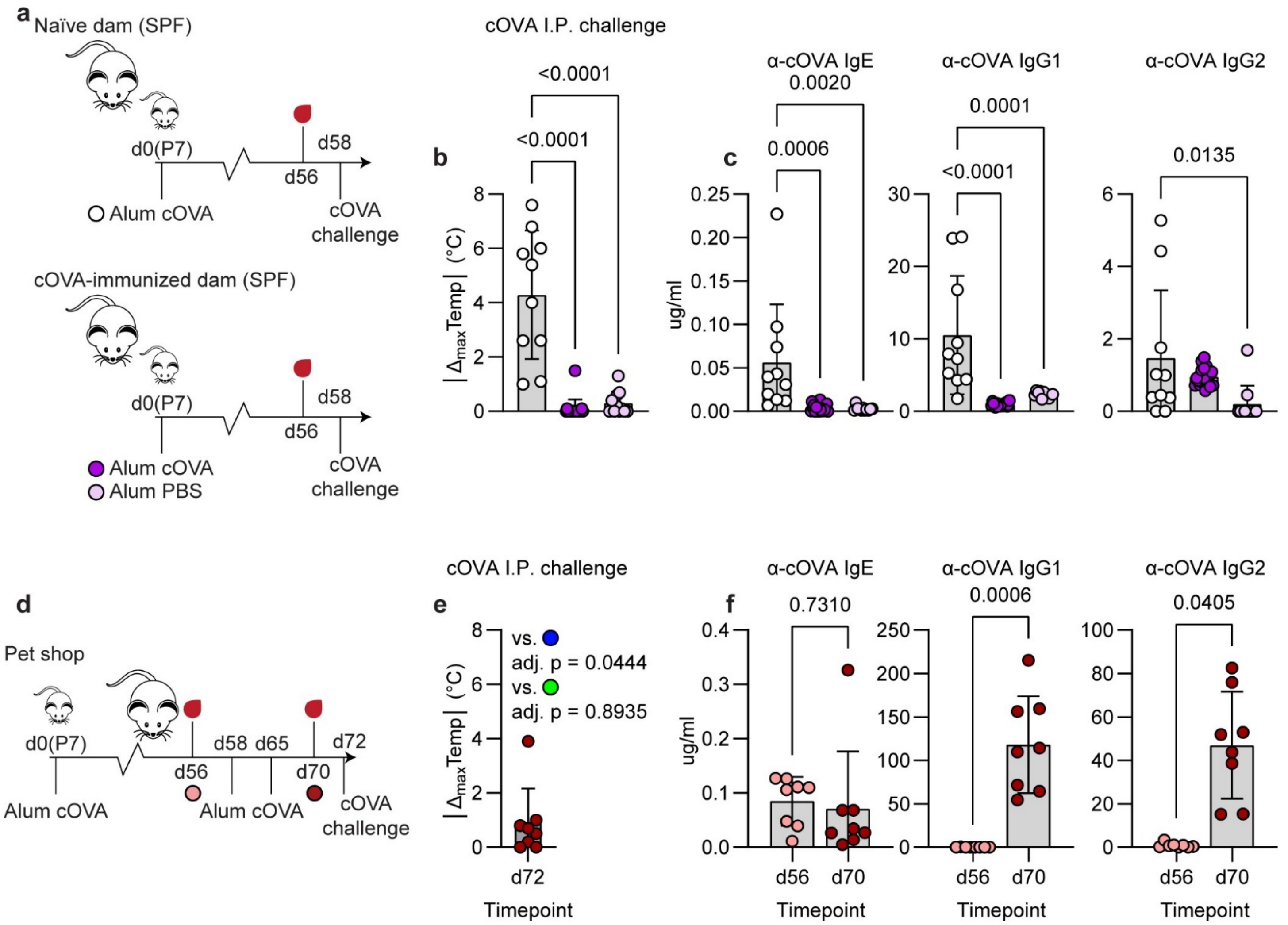

Supplemental Figure 6. Early life sensitization in pet shop mice and SPF mice born to immune dams. (**a**) Experimental scheme. P7 neonates from naïve or cOVA-immunized dams were subcutaneously injected with cOVA adsorbed to alum or alum alone, then bled at P63 (d56). Mice were challenged at P65 (d58). (**b**) Maximal temperature loss following challenge and (**c**) cOVA-reactive serum antibodies of the indicated isotype (n = 10 p7/naïve, 19 p7/immune, 11 null/immune). (**d**) Experimental scheme. Pet shop mice were subcutaneously injected with cOVA adsorbed to alum at P7 (d0) and serum samples collected at P63 (d56). The same mice were again subcutaneously injected with cOVA/alum on days P65 (d58) and P72 (d65) and serum samples were collected at P77 (d70). Mice were challenged I.P. with cOVA on d72. (**e**) Maximal temperature loss following challenge and (**f**) cOVA-reactive serum antibodies of the indicated isotype (n = 8).Statistical significance was determined using one-way ANOVA with Tukey correction for multiple comparisons (b,e,f) or two-tailed paired Student's t-test (c) .

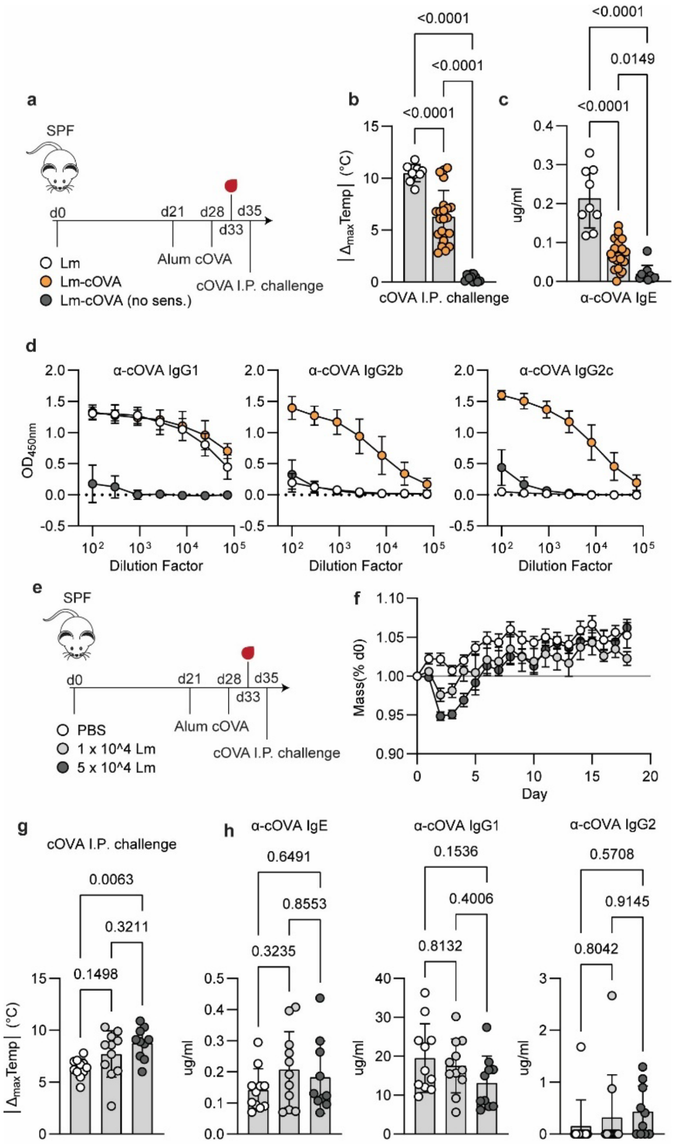

Supplemental Figure 7. Infection with cOVA-transgenic *Listeria monocytogenes* suppresses subsequent cOVA allergic sensitivity.

(**a**) Experimental scheme. SPF (C57BL/6J) mice were infected I.V. with 5x10^4 CFU of *Listeria monocytogenes* type strain 10403s (Lm) or *Lysteria monocytogenes* transgenically expressing cOVA (Lm-cOVA). Three weeks later, mice were subcutaneously sensitized with cOVA adsorbed to alum. Mice received an additional cOVA/alum injection one week later, and I.P. challenged after an additional week. Serum was collected two days prior to I.P. challenge. (**b**) Maximum core body temperature loss in mice previously infected with Lm or Lm-cOVA (n = 9 Lm, 22 Lm-cOVA). A group of Lm-cOVA-infected mice were spared post-infection cOVA exposures (no sens.) as an additional control (n= 10). (**c-d**) Measurement of serum cOVA-reactive antibodies of the indicated isotype (n = 4, representative of three experiments). (**e**) Experimental scheme. SPF (C57BL/6J) mice were infected I.V. with the indicated dose of Listeria monocytogenes type strain 10403s or left uninfected. Mice were then sensitized as in (a). (**f**) Mouse weight over time grouped by infectious dose. (**g**) Maximal temperature loss following cOVA I.P. challenge. (**h**) Measurement of serum cOVA-reactive antibodies of the indicated isotype. n = 11 PBS, 11 Lm $1x10^4$, 10 Lm $5x10^5$. Statistical significance was determined using one-way ANOVA with Tukey correction for multiple comparisons (b,c,g,h). Error bars represent SD (b,c,g,h) or SEM (d,f).

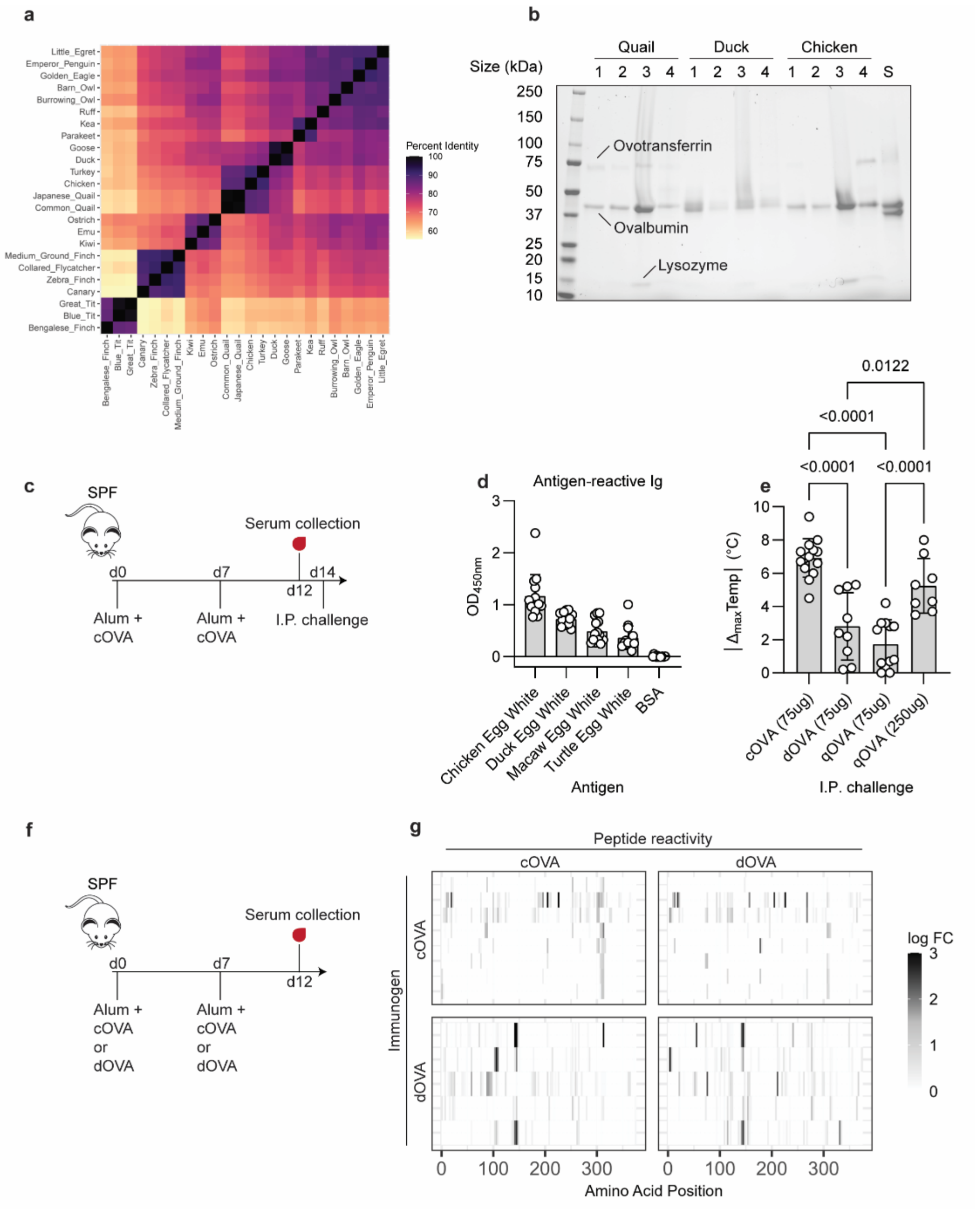

Supplemental Figure 8. Ovalbumin purification and serological cross-reactivity.

(**a**) Heatmap of ovalbumin protein sequence identity across avian species with annotated gene information (Ensembl). (**b**) Stain-free protein gel image of egg white fractionation. Lanes are labeled are as follows: 1, raw egg white; 2, PEG-rich fraction; 3, salt-rich fraction; 4, interface; S, grade V chicken ovalbumin (Sigma #A5503) (representative of five purifications). (**c**) Experimental scheme. SPF (C57BL/6J) mice were subcutaneously injected with cOVA adsorbed to alum on d0 and d7. Serum was collected on d12, and mice were I.P. challenged with cOVA or OVA orthologs on d14. (**d**) Measurement of cross-reactive serum antibodies from mice sensitized to cOVA (n = 14). (**e**) Maximum core body temperature loss in cOVA-sensitized mice systemically challenged with cOVA or OVA orthologs at the indicated doses (n = 14 cOVA/75, 9 dOVA/75, 12 qOVA/75, 8 qOVA/250). (**f**) Experimental scheme. SPF (C57BL/6J) mice were subcutaneously injected with cOVA or dOVA adsorbed to alum on d0 and d7. Serum was collected on d12 for epitope reactivity profiling. (**g**) Epitope profiling of cOVA and dOVA peptides against sera from mice immunized as in (f) (n = 8 cOVA-immunized, 5 dOVA-immunized). Statistical significance was determined using one-way ANOVA with Tukey correction for multiple comparisons (e). Error bars represent SD.

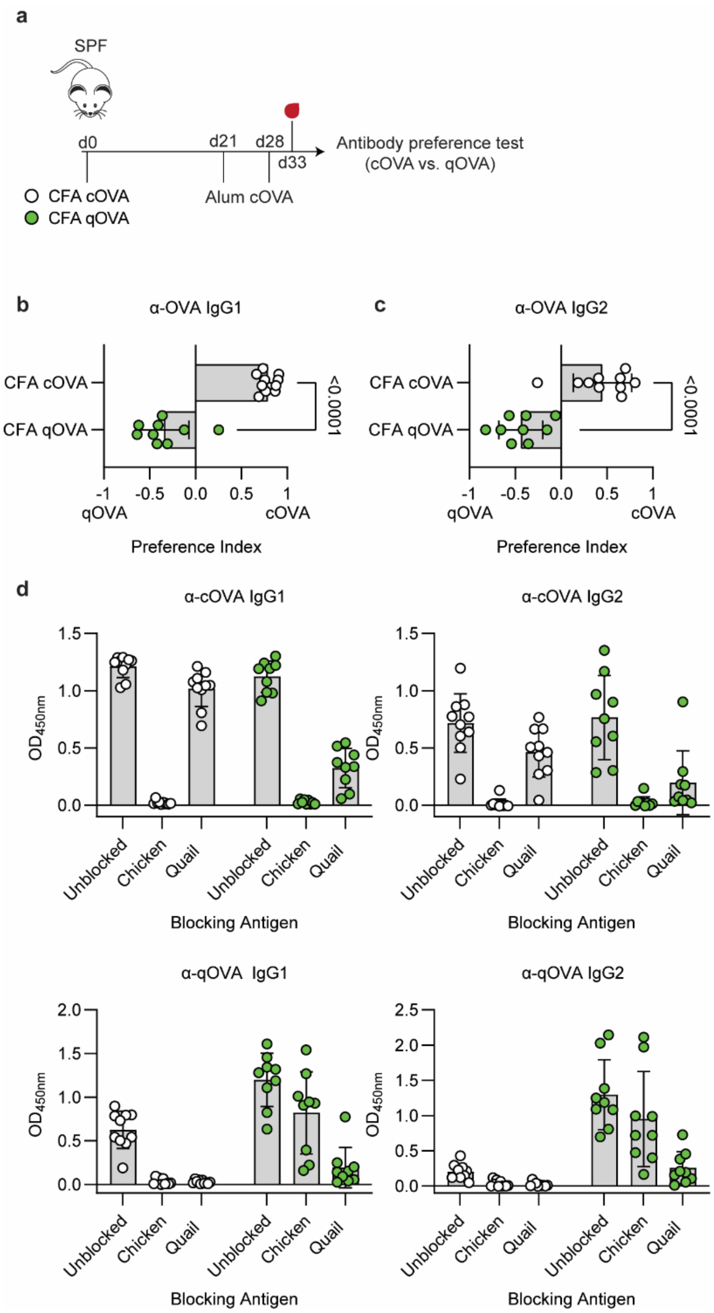

Supplemental Figure 9. Antibody binding preferences are established by primary immunization.

(**a**) Experimental scheme. (**b**,**c**) Preference index of serum IgG1 (**b**) and IgG2 (**c**) for cOVA or qOVA from SPF (C57BL/6J) mice of the indicated group (n = 10 cOVA/CFA, 9 qOVA/CFA). Serum at d33 from mice undergoing the indicated treatment were tested for reactivity to cOVA or qOVA in the presence or absence of soluble-phase cOVA or qOVA. Sera were normalized by dilution to ~5ng/ml cOVA IgG1 levels to allow for comparative measurement of blocking efficiency. A value of 1 indicates absolute preference for cOVA; a value of -1 indicates an absolute preference for qOVA. (**d**) Background-corrected OD_{450nm} values used for calculation of preference index. Statistical significance was determined using two-tailed unpaired Student's t-test (b-c). Error bars represent SD.

a

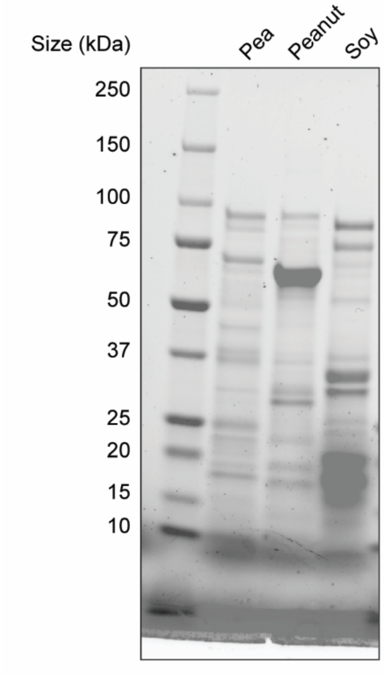

b

Soy protein	Ortholog (% identity)	
beta-conglycinin beta subunit 2	Pis s 1 (56.35%)	Pea
beta-conglycinin alpha subunit	Pis s 2 (54.91%)	Pea
non-specific lipid-transfer protein 3-like	Pis s 3 (62.10%)	Pea
putative pathogenesis-related protein	Pis s 6 (80.18%)	Pea
beta-conglycinin alpha subunit	Ara h 1 (41.33%)	Peanut
napin-type 2S albumin 1	Ara h 2 (36.71%)	Peanut
glycinin G3	Ara h 3 (57.99%)	Peanut
profilin	Ara h 5 (89.31%)	Peanut
hypothetical protein JHK85_038335	Ara h 6 (33.93%)	Peanut
2S seed storage albumin protein	Ara h 7 (31.21%)	Peanut
SRPBCC ligand-binding domain-containing protein	Ara h 8 (60.65%)	Peanut
hypothetical protein JHK85_001682	Ara h 9 (69.57%)	Peanut
P24 oleosin isoform A	Ara h 10 (58.47%)	Peanut
putative oleosin	Ara h 11 (75.76%)	Peanut
defensin-like protein	Ara h 12 (66.67%)	Peanut
defensin-like protein	Ara h 13 (57.33%)	Peanut
hypothetical protein JHK82_052064	Ara h 14 (49.17%)	Peanut
oleosin family protein	Ara h 15 (65.29%)	Peanut
hypothetical protein GYH30_051584	Ara h 16 (72.63%)	Peanut
hypothetical protein JHK85_001682	Ara h 17 (64.49%)	Peanut

Supplemental Figure 10. Preparation of legume extracts and antigen correspondence between soy and pea or peanut.

(**a**) Stain-free protein gel image of water-soluble legume extracts used for cross-reactivity experiments. (**b**) Antigen pairs between soy and pea or peanut for described allergenic proteins. Pea or peanut protein sequences were scanned against the soy proteome using p-BLAST and the nearest ortholog was listed along with overlap (as % identity).

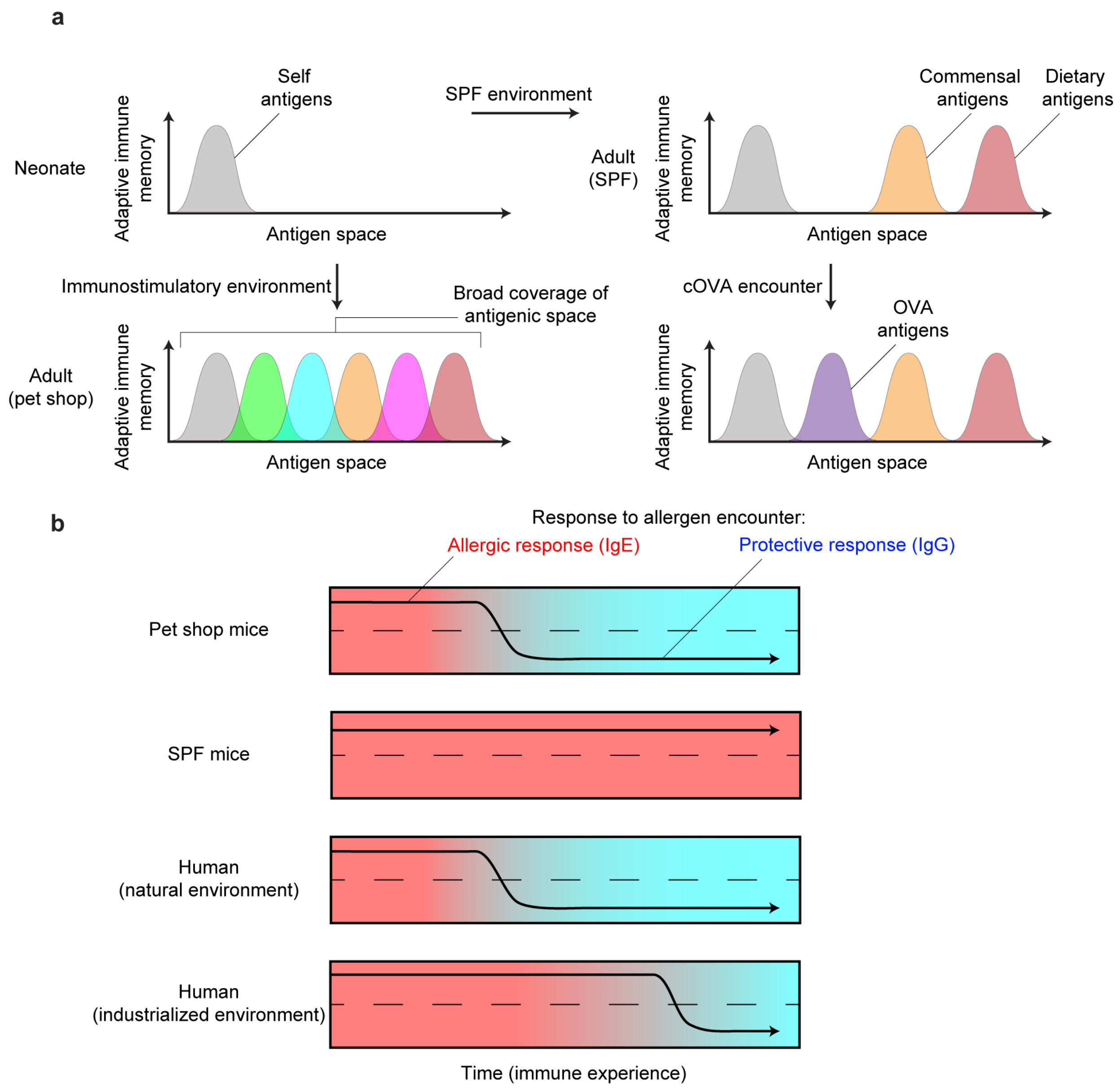

Supplemental Figure 11. Environmentally driven immune imprinting protects against allergy. (**a**) Representation of the antigen universe and breadth of adaptive immune recognition. Neonates have little antigen experience (adaptive immune memory, of any type) and will mount pure de novo adaptive immune responses against any foreign antigen. Different environmental life histories will cover varying areas of antigenic space; SPF mice may still mount de novo immune responses to model antigens, while pet shop mice are not truly naïve to many, if not all, foreign antigens. (**b**) The temporal window of allergic susceptibility. Antigen-rich environments stimulate the generation of cross-reactive adaptive immune memory, limiting sensitization potential. The duration of the susceptibility window is a function of antigen experience and may be prolonged in abnormal environmental conditions (SPF mice, humans in industrialized countries).

Supplemental Table 1. Pathogens detected in pet shop mice upon arrival at laboratory facilities.

**Positive Test**
Aspiculuris tetraptera
Campylobacter jejuni
Clostridium piliforme
Corynebacterium sp. (HAC2)
Cryptosporidium spp.
Demodex musculi
Entamoeba muris
Giardia muris
Helicobacter bilis
Helicobacter ganmani
Helicobacter mastomyrinus
Helicobacter spp.
Helicobacter typhlonius
MAV1
MAV2
MAD2
MHV
MNV
Mouse kidney parvovirus
MPV
MVM
Mycoplasma pulmonis
Myocoptes
Radfordia/Myobia
Rodentibacter pneumotropicus
Rodentolepis nana
Spironucleus muris
Staphylococcus aureus
Staphylococcus xylosus
Syphacia obvelata
TMEV GDVII
Tritrichomonas muris

Supplemental Table 2. Pathogens detected in SPF-fostered pet shop mice

**Positive Test**
Helicobacter spp.
MNV
Spironucleus muris
Tritrichomonas muris

Supplemental Table 3. Pathogens detected in pet shop-fostered and co-housed inbred mice

**Positive Test**
Corynebacterium sp. (HAC2)
Giardia muris
Helicobacter ganmani
Helicobacter mastomyrinus
Helicobacter spp.
Helicobacter typhlonius
MAD2
MNV
Myocoptes
Radfordia/Myobia
Rodentibacter pneumotropicus
Staphylococcus xylosus
Syphacia obvelata

Supplemental Table 4. Antibodies used for ELISA

Antibody Target	Clone	Catalog #	Source
IgA	Polyclonal (goat)	1040-01	Southern Biotech
IgA (standard)	M18-254	553476	BD
IgE	R35-72	553413	BD
IgE	Polyclonal (goat)	1110-05	Southern Biotech
IgG1	Polyclonal (goat)	1071-01/1071-05	Southern Biotech
IgG2a	Polyclonal (goat)	1081-01/1081-05	Southern Biotech
IgG2b	Polyclonal (goat)	1091-01/1091-05	Southern Biotech
IgG2c	Polyclonal (goat)	1078-01/1078-05	Southern Biotech
IgG3	Polyclonal (goat)	1101-01	Southern Biotech
IgG3 (standard)	A112-3	553486	BD
IgM	Polyclonal (goat)	1021-01	Southern Biotech
IgM (standard)	C48-6	557275	BD
Mouse kappa chain	Polyclonal (goat)	1050-05	Southern Biotech
Mouse lambda chain	Polyclonal (goat)	1060-05	Southern Biotech
OVA-IgE (standard)	E-C1	3006	Chondrex
OVA-IgG1 (standard)	2322	7094	Chondrex
OVA-IgG2a (standard)	M12E4D5	7095	Chondrex
OVA-IgG2b (standard)	4B4E6	7096	Chondrex
OVA-IgG2c (standard)	3E3A9	7109	Chondrex

Supplemental Table 5. Antibodies used for flow cytometry

Antibody Target	Clone	RRID	Source
CD154	MR1	AB_313268	Biolegend
CD4	GK1.5	AB_2738426	BD
CD44	IM7	AB_2564214	Biolegend
CD45	30-F11	AB_2875194	BD
CD62L	MEL-14	AB_313089	Biolegend
CD8a	53-6.7	AB_312744	Biolegend
IFNy	XMG1.2	AB_395376	BD
IL-4	BVD6-24G2	AB_468413	BD
TCRb	H57-597	AB_10933263	Biolegend
