# Supplementary material for: Environmentally driven immune imprinting protects against allergy
Source: Nature. 2026 Jan 28;650(8103):987–96. doi: 10.1038/s41586-025-10001-5 (PMC12935535; doi:10.1038/s41586-025-10001-5)
Supplement: Supplementary file 2 — Reporting Summary [file 41586_2025_10001_MOESM2_ESM.pdf]

nature portfolio

Corresponding author(s): Ruslan Medzhitov

Last updated by author(s): Nov 17, 2025

Reporting Summary

Nature Portfolio wishes to improve the reproducibility of the work that we publish. This form provides structure for consistency and transparency in reporting. For further information on Nature Portfolio policies, see our Editorial Policies and the Editorial Policy Checklist.

Statistics

For all statistical analyses, confirm that the following items are present in the figure legend, table legend, main text, or Methods section.

n/a	Confirmed	
☐	☒	The exact sample size (*n*) for each experimental group/condition, given as a discrete number and unit of measurement
☐	☒	A statement on whether measurements were taken from distinct samples or whether the same sample was measured repeatedly
☐	☒	The statistical test(s) used AND whether they are one- or two-sided<br>*Only common tests should be described solely by name; describe more complex techniques in the Methods section.*
☒	☐	A description of all covariates tested
☐	☒	A description of any assumptions or corrections, such as tests of normality and adjustment for multiple comparisons
☐	☒	A full description of the statistical parameters including central tendency (e.g. means) or other basic estimates (e.g. regression coefficient) AND variation (e.g. standard deviation) or associated estimates of uncertainty (e.g. confidence intervals)
☐	☒	For null hypothesis testing, the test statistic (e.g. *F*, *t*, *r*) with confidence intervals, effect sizes, degrees of freedom and *P* value noted<br>*Give P values as exact values whenever suitable.*
☒	☐	For Bayesian analysis, information on the choice of priors and Markov chain Monte Carlo settings
☒	☐	For hierarchical and complex designs, identification of the appropriate level for tests and full reporting of outcomes
☒	☐	Estimates of effect sizes (e.g. Cohen's *d*, Pearson's *r*), indicating how they were calculated

Our web collection on statistics for biologists contains articles on many of the points above.

Software and code

Policy information about availability of computer code

Data collection	Data collection for flow cytometry was performed with BD Facsdiva (v8.0.1). Data collection for ELISA was performed with Molecular Devices SoftMax Pro. Data collection for ELISpot was performed with Autoimmun Diagnostika GMBH AID. Data collection for next-generation sequencing was performed with Illumina BCLConvert.
Data analysis	R (v4.3), phippery (v.1.2.0), Graphpad Prism (v10.2.3), FlowJo (v10.8.1).

For manuscripts utilizing custom algorithms or software that are central to the research but not yet described in published literature, software must be made available to editors and reviewers. We strongly encourage code deposition in a community repository (e.g. GitHub). See the Nature Portfolio guidelines for submitting code & software for further information.

Data

Policy information about availability of data

All manuscripts must include a data availability statement. This statement should provide the following information, where applicable:

- Accession codes, unique identifiers, or web links for publicly available datasets
- A description of any restrictions on data availability
- For clinical datasets or third party data, please ensure that the statement adheres to our policy

All data are available on request from the corresponding author.

Research involving human participants, their data, or biological material

Policy information about studies with human participants or human data. See also policy information about sex, gender (identity/presentation), and sexual orientation and race, ethnicity and racism.

Reporting on sex and gender	n/a
Reporting on race, ethnicity, or other socially relevant groupings	n/a
Population characteristics	n/a
Recruitment	n/a
Ethics oversight	n/a

Note that full information on the approval of the study protocol must also be provided in the manuscript.

Field-specific reporting

Please select the one below that is the best fit for your research. If you are not sure, read the appropriate sections before making your selection.

☒ Life sciences ☐ Behavioural & social sciences ☐ Ecological, evolutionary & environmental sciences

For a reference copy of the document with all sections, see nature.com/documents/nr-reporting-summary-flat.pdf

Life sciences study design

All studies must disclose on these points even when the disclosure is negative.

Sample size	Sample sizes were chosen to give a minimum of n = 5 across a minimum of two independent experiments.
Data exclusions	No data were excluded.
Replication	Experiments were performed at least twice; all attempts at replication were successful.
Randomization	Mice were randomly assigned to experimental groups.
Blinding	Investigators were not blinded to group allocation during data collection as these experiments were performed by individual investigators who were aware of groups and treatments.

Reporting for specific materials, systems and methods

We require information from authors about some types of materials, experimental systems and methods used in many studies. Here, indicate whether each material, system or method listed is relevant to your study. If you are not sure if a list item applies to your research, read the appropriate section before selecting a response.

Materials & experimental systems

n/a	Involved in the study	
☐	☒	Antibodies
☐	☒	Eukaryotic cell lines
☒	☐	Palaeontology and archaeology
☐	☒	Animals and other organisms
☒	☐	Clinical data
☒	☐	Dual use research of concern
☒	☐	Plants

Methods

n/a	Involved in the study	
☒	☐	ChIP-seq
☐	☒	Flow cytometry
☒	☐	MRI-based neuroimaging

Antibodies

Antibodies used

Antibody Target Clone RRID Source
1. CD154 MR1 AB_313268 Biolegend
2. IL-4 BVD6-24G2 AB_468413 BD

3. IFNy XMG1.2 AB_395376 BD
4. CD62L MEL-14 AB_313089 Biolegend
5. CD8a 53-6.7 AB_312744 Biolegend
6. CD44 IM7 AB_2564214 Biolegend
7. TCRb H57-597 AB_10933263 Biolegend
8. CD45 30-F11 AB_2875194 BD
9. CD4 GK1.5 AB_2738426 BD

Validation

1. https://www.biolegend.com/de-at/products/biotin-anti-mouse-cd154-antibody-404
2. https://www.thermofisher.com/antibody/product/IL-4-Antibody-clone-BVD6-24G2-Monoclonal/11-7042-82
3. https://www.bdbiosciences.com/en-us/products/reagents/flow-cytometry-reagents/research-reagents/single-color-antibodies-ruo/pe-rat-anti-mouse-ifn.562020
4. https://www.biolegend.com/de-de/products/percp-anti-mouse-cd62l-antibody-4273
5. https://www.biolegend.com/en-gb/products/fitc-anti-mouse-cd8a-antibody-153?GroupID=BLG2559
6. https://www.biolegend.com/fr-ch/products/brilliant-violet-711-anti-mouse-human-cd44-antibody-10316
7. https://www.biolegend.com/en-ie/products/brilliant-violet-421-anti-mouse-tcr-beta-chain-antibody-7251?GroupID=BLG6994
8. https://www.bdbiosciences.com/en-us/products/reagents/flow-cytometry-reagents/research-reagents/single-color-antibodies-ruo/buv615-rat-anti-mouse-cd45.751170
9. https://www.bdbiosciences.com/en-us/products/reagents/flow-cytometry-reagents/research-reagents/single-color-antibodies-ruo/buv395-rat-anti-mouse-cd4.565974

Eukaryotic cell lines

Policy information about cell lines and Sex and Gender in Research

Cell line source(s): Expi293 (Thermofisher #A14635)

Authentication: No cell line authentication was performed.

Mycoplasma contamination: Cell line not tested for mycoplasma.

Commonly misidentified lines (See ICLAC register): n/a

Animals and other research organisms

Policy information about studies involving animals; ARRIVE guidelines recommended for reporting animal research, and Sex and Gender in Research

Laboratory animals: Pet shop mice were purchased from Komodo Reptile, LLC (Verplanck, NY). Mice of the following strains were purchased from The Jackson Laboratory (Bar Harbor, ME): 129S1/SvImJ (Strain #002448), A/J (#000646), BALB/cJ (#000651), CAST/EiJ (#000928), C3H/HeJ (#000659), C57BL/6J (#000664), DBA/1J (#000670), FVB/NJ (#001800), PERC/EiJ (#001307), PWK/PhJ (#003715) SJL/J (#000686), and WSB/EiJ (#001145). All mice were 8-12 weeks at initiation of experiments.

Wild animals: n/a

Reporting on sex: Both male and female mice were used in experiments requiring in-house breeding of cohorts. In other experiments, female mice were used.

Field-collected samples: n/a

Ethics oversight: All protocols were reviewed, approved, and conducted under the institutional regulation of Yale University's Institutional Animal Care and Use Committee.

Note that full information on the approval of the study protocol must also be provided in the manuscript.

Plants

Seed stocks: n/a

Novel plant genotypes: n/a

Authentication: n/a

Flow Cytometry

Plots

Confirm that:

☒ The axis labels state the marker and fluorochrome used (e.g. CD4-FITC).

☒ The axis scales are clearly visible. Include numbers along axes only for bottom left plot of group (a 'group' is an analysis of identical markers).

☒ All plots are contour plots with outliers or pseudocolor plots.

☒ A numerical value for number of cells or percentage (with statistics) is provided.

Methodology

Sample preparation	Splenic single cell suspensions prepared as described above were incubated at 1x107 cells/ml in 24-well plates (Falcon #353047) with complete RPMI +/- 100ug/ml antigen in incubators kept at 37deg.c, 5% CO2, 95% RH. After six hours of incubation, Brefeldin A (Sigma #B7651) was added to wells followed by a further 12-hour incubation. Cells were removed from wells and incubated with aCD16/CD32 (Biolegend #101320). Surface staining was performed simultaneously with Zombie UV (Biolegend #423107). After surface staining, cells were washed and fixed (BD #554714) according to manufacturer's specifications and stained for intracellular antigens.<br>For bacterial flow cytometry, samples were prepared as described for peptide enrichment. Following serum/mAb incubation, samples were stained with FITC-conjugated polyclonal goat anti-mouse IgG (Southern Biotech #103302) in PBST.
Instrument	BD Symphony or BD LSRII
Software	Facsdiva (BD)
Cell population abundance	No cell sorting was performed.
Gating strategy	Gates are shown where relevant. Cells were gated by size, as single cells, as live/CD45+, then for specific T cell markers.<br>Bacteria were gated by size, then by antibody binding.

☒ Tick this box to confirm that a figure exemplifying the gating strategy is provided in the Supplementary Information.
